# Loss-of-function mutations in *QRICH2* cause male infertility with multiple morphological abnormalities of the sperm flagella

**DOI:** 10.1038/s41467-018-08182-x

**Published:** 2019-01-25

**Authors:** Ying Shen, Feng Zhang, Fuping Li, Xiaohui Jiang, Yihong Yang, Xiaoliang Li, Weiyu Li, Xiang Wang, Juan Cheng, Mohan Liu, Xueguang Zhang, Guiping Yuan, Xue Pei, Kailai Cai, Fengyun Hu, Jianfeng Sun, Lanzhen Yan, Li Tang, Chuan Jiang, Wenling Tu, Jinyan Xu, Haojuan Wu, Weiqi Kong, Shuying Li, Ke Wang, Kai Sheng, Xudong Zhao, Huanxun Yue, Xiaoyu Yang, Wenming Xu

**Affiliations:** 1 Department of Obstetrics/Gynecology, Joint Laboratory of Reproductive Medicine (SCU-CUHK), Key Laboratory of Obstetric, Gynecologic and Pediatric Diseases and Birth Defects of Ministry of Education, West China Second University Hospital, Sichuan University, 610041 Chengdu, China; 2 Obstetrics and Gynecology Hospital, Institute of Metabolism and Integrative Biology, School of Life Sciences, Fudan University, 200011 Shanghai, China; 3 State Key Laboratory of Reproductive Medicine, Center for Global Health, School of Public Health, Nanjing Medical University, 211116 Nanjing, China; 4 Shanghai Key Laboratory of Female Reproductive Endocrine Related Diseases, 200011 Shanghai, China; 5 Human Sperm Bank, West China Second University Hospital, Sichuan University, 610041 Chengdu, China; 6 Key Laboratory of Birth Defects and Related Disease of Women and Children (Sichuan University), Ministry of Education, 610041 Chengdu, China; 7 Center of Reproductive Medicine, West China Second University Hospital, Sichuan University, 610041 Chengdu, China; 8 Southwest Medical University, 646000 Luzhou, China; 9 Department of Medical Genetics, State Key Laboratory of Biotherapy, West China Hospital, Sichuan University and Collaborative Innovation Center, 610041 Chengdu, China; 10 Analytical & Testing Center, Sichuan University, 610041 Chengdu, China; 11 Chengdu Research Base of Giant Panda Breeding, 610041 Chengdu, China; 12 Auckland University, 1142 Auckland, New Zealand; 13 Teaching Hospital of Chengdu University of TCM, 610041 Chengdu, China; 14 Key Laboratory of Animal Models and Human Disease Mechanisms of Chinese Academy of Sciences and Yunnan Province, Kunming Institute of Zoology, Chinese Academy of Sciences, 650000 Kunming, China; 15 Center for Excellence in Animal Evolution and Genetics, Chinese Academy of Sciences, 650223 Kunming, China; 16 State Key Laboratory of Reproductive Medicine, Clinical Center of Reproductive Medicine, The First Hospital, Nanjing Medical University, 210029 Nanjing, China

**Keywords:** Genetics, CRISPR-Cas systems, Medical genetics, Next-generation sequencing

## Abstract

Aberrant sperm flagella impair sperm motility and cause male infertility, yet the genes which have been identified in multiple morphological abnormalities of the flagella (MMAF) can only explain the pathogenic mechanisms of MMAF in a small number of cases. Here, we identify and functionally characterize homozygous loss-of-function mutations of *QRICH2* in two infertile males with MMAF from two consanguineous families. Remarkably, *Qrich2* knock-out (KO) male mice constructed by CRISPR-Cas9 technology present MMAF phenotypes and sterility. To elucidate the mechanisms of *Qrich2* functioning in sperm flagellar formation, we perform proteomic analysis on the testes of KO and wild-type mice. Furthermore, in vitro experiments indicate that QRICH2 is involved in sperm flagellar development through stabilizing and enhancing the expression of proteins related to flagellar development. Our findings strongly suggest that the genetic mutations of human *QRICH2* can lead to male infertility with MMAF and that *QRICH2* is essential for sperm flagellar formation.

## Abstract (web summary)

Multiple morphological abnormalities of the sperm flagella (MMAF) is a cause of male infertility. Here the authors identify homozygous nonsense mutations of the *glutamine rich 2* (*QRICH2*) gene in two MMAF patients from 2 consanguineous families and show using *QRICH2* knockout mice that the protein is required for sperm flagellar formation and motility.

## Introduction

Sperm defects are the direct causes of male infertility, which includes decreased sperm count, reduced sperm motility and abnormal morphology^1,2^. Sperm motility is a critical factor for normal fertilization, and over 80% of male infertility is caused by impaired sperm motility^3^. Sperm morphology plays an essential role in sperm locomotion, and defective sperm morphology makes an assignable contribution to male infertility, which consists of a wide range of phenotypes involving the head, neck, mid-piece or tail of the spermatozoa. Among these, multiple morphological abnormalities of the sperm flagella (MMAF) is characterized by a mosaic of morphological abnormalities of the flagellum including coiled, bent, irregular, short or/and absent flagella^4–6^. Normal sperm flagella contain a 9 + 2 axonemal arrangement of nine peripheral microtubule doublets (OD) and two central microtubules (CP), surrounded by outer dense fiber (ODF) and fibrous sheath (FS)^7^. Almost all MMAF cases are accompanied by visible ultrastructural derangement and/or defects, such as incomplete or disorganized axoneme arrangement or absence of ODF/FS^5,6,8^. Any defects in these structures would result in aberrant sperm morphology and further lead to reduced motility or even sperm immobility.

Research is ongoing to investigate the molecular mechanisms of MMAF. Using knock-out (KO) mouse models, several genes have been found to be associated with sperm flagellar development in mice^9^. The complete ablation of *Spata6* caused impaired development of the connecting piece and tight head-tail conjunction^10^. Subtle disorganization of the ultrastructure was observed in *Tekt4*-null mice^11^. The sperm flagella of *Tssk4*-absent mice represented morphological defects at the midpiece-principal piece junction^12^. Mice lacking *Odf2* showed axoneme defects^13^. *Ropn1*-deficient mice exhibited structural defects in the principal piece of sperm flagellum^14^. A significant disorganization in the fibrous sheath, as well as abnormal configuration of doublet microtubules, was observed in *Cabyr*-knockout male mice^15^. Although MMAF is not a rare autosomal-recessive inherited disorder in humans, the genetic etiology of a large proportion of MMAF cases remains elusive^4–6^. To date, mutations in only six genes have been determined in humans related to MMAF: *AKAP4*, *CCDC39*, *DNAH1*, *CFAP43*, *CFAP44*, and *CFAP69* (refs. ^4,5,8,16–19^); however, these could explain the mechanism of only a few MMAF cases. Therefore, it remains necessary to improve the discriminative power of a single candidate gene in human MMAF.

In this study, two homozygous nonsense mutations of the glutamine rich 2 (*QRICH2*) gene are identified in two MMAF patients from two consanguineous families through whole-exome sequencing (WES). The *Qrich2* KO mouse model represents typical MMAF phenotypes including coiled, bent, irregular, short or/and absent flagella and defects of sperm flagellar ultrastructure. These phenotypes are consistent with those observed in human subjects who carried the loss-of-function mutations of *QRICH2*. This study provides compelling evidence that the loss-of-function mutations of *QRICH2* could result in MMAF and cause male infertility, and QRICH2 is a functional molecule essential for sperm flagellar development by regulating the genes associated with the accessory structure of sperm flagella.

## Results

### Loss-of-function mutations of *QRICH2* in males with MMAF

**Fig. 1:**
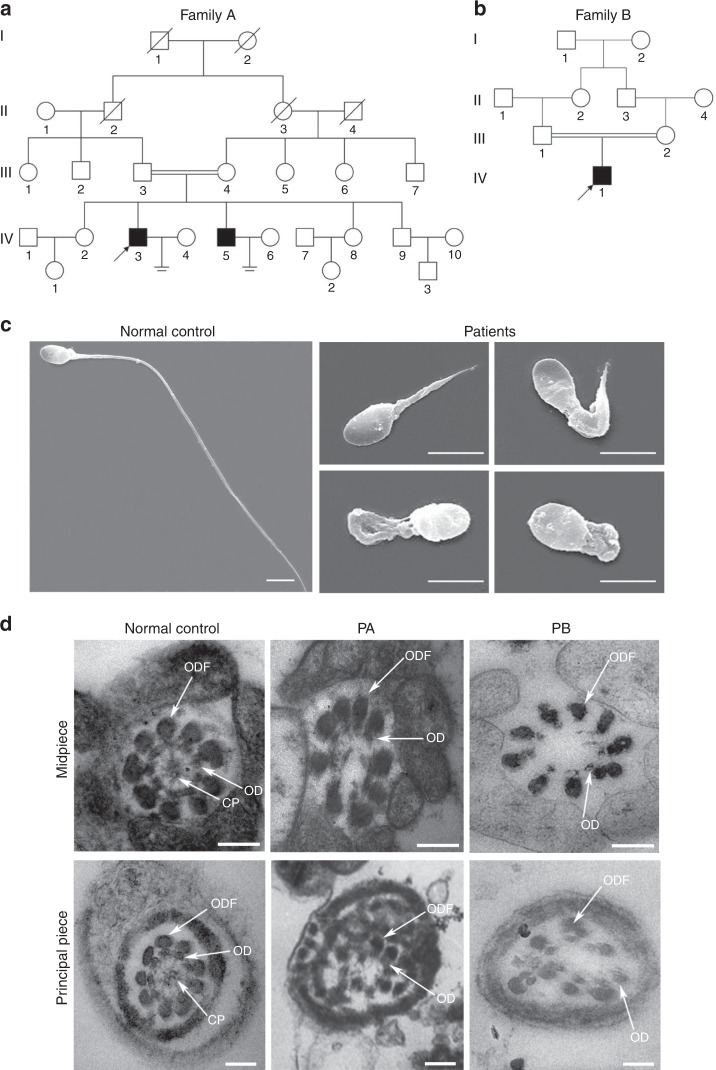
Sperm morphology and ultrastructure in the two MMAF patients. **a**, **b** The pedigree structure and segregation analysis in two families. Squares represent male pedigree members, circles represent female pedigree members, solid symbols represent members with MMAF, and open symbols represent unaffected members; the probands are indicated by black arrows. **c** The malformations of sperm flagella in the two MMAF individuals. The absent, short, thick, or coiled flagella and other MMAF phenotypes were observed in two patients compared with the normal control by SEM (scale bars, 5 µm). **d** The abnormal ultrastructures of the mid-piece and principal piece of sperm flagella in two patients. By TEM, in the mid-piece, PA exhibited an atypical 9 + 0 arrangement of axonemal microtubules, and the CP and OD defects were observed in PB. The principal piece of the PA and PB flagella consist of incomplete and disorganized OD and ODF and lack CP. Fibrous sheaths were not apparent, especially for PB. (scale bars, 100 nm)

Two infertile males from two consanguineous families were investigated in our study (Fig. 1a, b). Semen analysis is presented in Table 1. The sperm quantity was basically normal, whereas abnormalities in the spermatozoa tail region were up to 99%. Therefore, almost no progressive motility of spermatozoa was observed in these two cases. Scanning electron microscopy (SEM) further identified the MMAF phenotypes of these two cases. We observed some spermatozoa with short tails, some with thick short tails, and some with coiled tails, and some spermatozoa appeared to have only the head region (Fig. 1c). In addition, a variety of ultrastructural defects were detected in the sperm flagella under transmission electron microscopy (TEM) (Fig. 1d). For patient A (PA) integrated and regularly arranged OD and ODF were observed, whereas the CP were missed in the mid-piece of most flagella. In the principal piece of most flagella, some ODF and OD were absent, the remainders were disorganized, and the CP could not be observed as well. For patient B (PB) the atypical 6 + 0 composition of axonemal microtubules was shown in the flagellar mid-piece; additionally, a complete lack of CP and the irregular arrangement of the OD and ODF were revealed in the principal piece.

**Table 1:** Semen and variant analysis in the MMAF patients from consanguineous families

			Patient A	Patient B
Semen parameters	Semen volume (ml)		5	4
	Semen concentration (10^6^/ml)		35	14.4
	Progressive motility (%)		0	2.7
	Motility (%)		0	5.4
Sperm morphology	Normal flagella (%)		1	1
	Aberrant flagella (%)		99	99
	cDNA mutation^a^		c.192 G > A	c.3037 C > T
	Protein alteration		p. L64^a^	p.R1013^a^
	Variant type		nonsense	nonsense
	Allele frequency			
Variant	ExAC Browser	Total	0	0.00002471
		East Asians	0	0
	GnomAD	Total	0	0.00001989
		East Asians	0	0
	1000 Genomes Project	Total	0	0
		East Asians	0	0
	In-house Chinese-control		0	0

Aberrant flagella mainly represent morphological abnormalities of the flagellum including coiled, bent, irregular, short or/and absent flagella

^a^The accession number for *QRICH2* is GenBank: NM_032134.2

The previous studies have reported that the homozygous loss-of-function mutations of several genes could cause MMAF and have suggested that MMAF is a disorder with an autosomal-recessive inheritance pattern^5,8,16,19^. According to this, we performed WES on the two males to elucidate the underlying genetic causes of the MMAF phenotypes, and many homozygous variants were found in the two patients (Supplementary Data 1). Briefly, variants were removed if (1) the minor allele frequency was ≥1% in any database, including the gnomAD, ExAC Browser and 1000 Genomes Project, because the pathogenic variants responsible for MMAF are rare in human populations; (2) the heterozygous variant, because MMAF has been assumed to be an autosomal-recessive inheritance; (3) the variant affected: 3′ or 5′ untranslated regions, noncoding exons, or intronic sequences except canonical splice sites; and (4) the variant was not predicted damaging by PolyPhen-2, SIFT and MutationTaster tools. Subsequently, the selected genes harboring candidate pathogenic variants from WES were evaluated by Human Protein Atlas (http://www.proteinatlas.org), which contains tissue-specific protein expression data, and the genes which are primarily or specifically expressed in the testis were included in our research. Remarkably, after exclusion of frequent variants and application of technical and biological filters, two homozygous nonsense mutations were identified in *QRICH2* (Table 1): a homozygous stop-gain mutation in exon 3 (c.192G>A [p. L64*]) in the individual PA and a homozygous stop-gain variant in exon 4 in the individual PB (c.3037C>T [p.R1013*]). To clarify the putative contribution of these mutations to the MMAF phenotypes, we investigated the *QRICH2* gene by Sanger sequencing in PA and another affected individual (IV:3 and IV:5), their healthy parents (III:3 and III:4), and unaffected siblings (IV:2, IV:8 and IV:9) from Family A. In this family, the another affected individual (IV:5) carried the homozygous mutation (c.192G>A) and their healthy parents (III:3 and III:4), as well as one unaffected sibling (IV:8) carried the heterozygous mutation (c.192G>A) (Supplementary Fig. 1a). For family B, we detected the heterozygous mutation (c.3037C>T) in their healthy parents (III:1 and III:2) (Supplementary Fig. 1b). The results of DNA sequencing suggested that the two homozygous nonsense mutations of PA and PB were inherited from their parents carrying heterozygous mutations, which is in line with the nature of MMAF being an autosomal recessive inheritance.

### The expression of *QRICH2* in sperm flagella

**Fig. 2:**
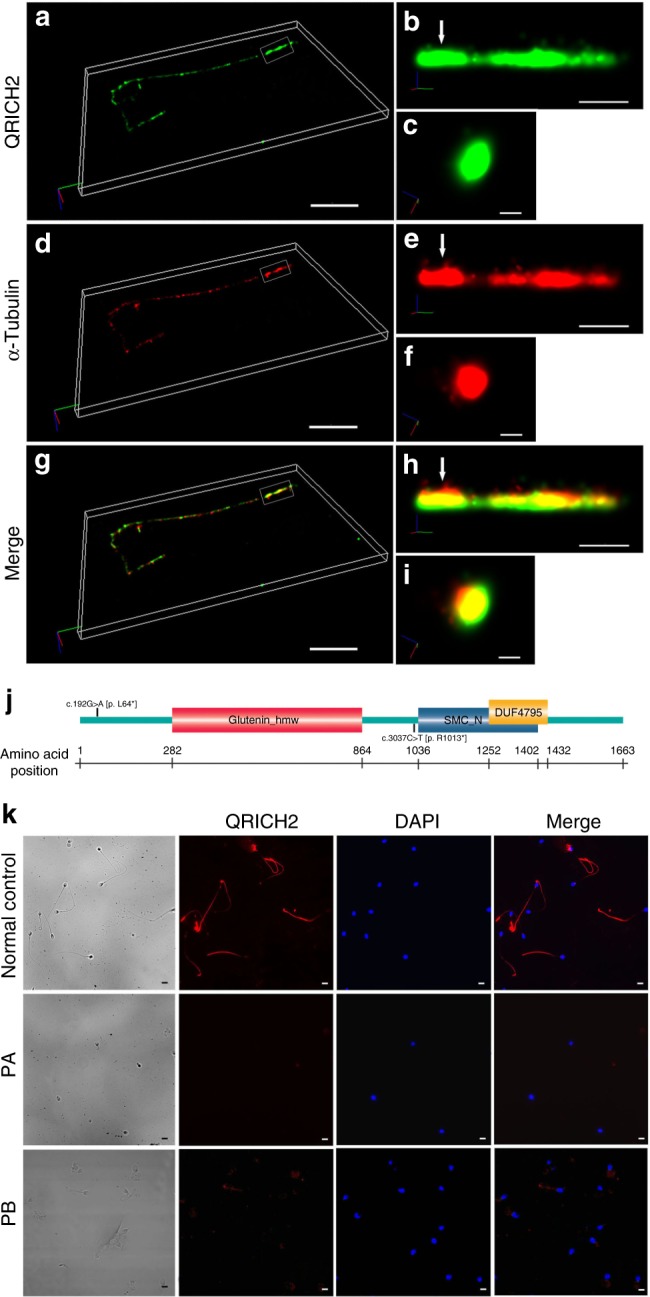
QRICH2 is detected in human sperm flagella. **a**–**i** 3D STORM images of QRICH2 (green) (**a**–**c**), α-Tubulin (red) (**d**–**f**) and their co-localization (yellow) (**g**–**i**). **a**, **d**, **g**, *x*-*y*-*z* projections (scale bars, 5 µm). **b**, **e**, **h**, *x*–*y* projections: sections surrounded by white square (scale bars, 0.5 µm). **c**, **f**, **i**, y-z cross-sections near the annulus (white arrowheads) (scale bars, 0.1 µm). **j** The domains of QRICH2 and the positions of two mutations in *QRICH2*. QRICH2 contains three domains: Glutenin hmw super family (amino acid positions 282–864), SMC_N super family (amino acid positions 1036–1402) and DUF4795 (amino acid positions 1252–1432). **k** Immunofluorescence staining of QRICH2 on human spermatozoa from normal control and the two MMAF individuals. QRICH2 staining (red) was absent in sperm from PA and significantly weak in sperm from PB. (blue, DAPI; scale bars, 5 µm)

The Human Proteins Atlas Database indicates that the expression of human *QRICH2* is restricted to the testes, while no information was available with regard to QRICH2 protein distribution in spermatozoa. To determine the location of QRICH2 on human spermatozoa accurately, we used stochastic optical reconstruction microscopy (STORM), which enables 3D reconstruction at 10–20 nm or higher resolutions^20–23^. As expected, 3D STORM images of QRICH2 showed a continuous distribution along the sperm flagella (Fig. 2a–c, Supplementary Movie 1). Notably, QRICH2 co-localized with microtubule protein α-Tubulin which is an axonemal component, indicating that QRICH2 is expressed in sperm tail and plays a vital role in sperm flagellar development (Fig. 2d–i, Supplementary Movies 2, 3).

The *QRICH2* gene is localized on human chromosome 17, contains 19 exons and encodes a predicted 1663-amino acid protein (NP_115510.1) with three principal domains (Fig. 2j). The homozygous stop-gain mutation of c.192G>A [p. L64*] is located in exon 3 of *QRICH2* which causes premature translational termination of QRICH2 protein resulting in the absence of three domains of QRICH2 (Fig. 2d). The other homozygous stop-gain variant, c.3037C>T [p.R1013*], which is located in exon 4 of *QRICH2*, results in the loss of SMC_N super family domain and DUF4795 domain (Fig. 2j). To confirm the effect of the two mutations on *QRICH2* expression, we performed immunofluorescence assays on spermatozoa of the two MMAF patients. The QRICH2 protein was not detected in spermatozoa from PA and was remarkably weak in spermatozoa from PB (Fig. 2k). Thus, the two homozygous stop-gain variants did impact the expression of *QRICH2* negatively, leading to the abnormal development of sperm flagella.

### Lacking *Qrich2* leads to MMAF in male mice

*QRICH2* is highly conserved during evolution. The human QRICH2 shares 96.7, 89.5, 74.2, 61.2, 81.8, and 59.5% of protein identity with its orthologs in chimpanzees, rhesus monkeys, dogs, cattle, mice and rats, respectively (https://www.ncbi.nlm.nih.gov) (Supplementary Fig. 2a, b). The expression profile of *Qrich2* in adult mice suggested that *Qrich2* is primarily expressed in the testis tissue (Supplementary Fig. 2c). Accurately, immunofluorescent stain of testis sections from WT mice showed that Qrich2 is mainly expressed in the round and elongated spermatids during the spermiogenesis, and the Qrich2 staining is absent in the testis sections from KO mice (Supplementary Fig. 3a–d). To better understand the localization of Qrich2 in different steps of germ cells, we carried out flow cytometry coupled to cell sorting assay to isolate elongated spermatids, round spermatids and spermatogonia. The immunofluoresence results indicated that Qrich2 is distributed in the nuclear membrane of the spermatogonia (Supplementary Fig. 3e), in the nucleus of the round spermatids (Supplementary Fig. 3f), in the nucleus and cytoplasm of the early elongating spermatids (Supplementary Fig. 3g), in the cytoplasm of late elongating spermatids (Supplementary Fig. 3h) and in the flagella of epididymal spermatozoa (Supplementary Fig. 3i). Furthermore, to find out the temporal expression of *Qrich2*, *Qrich2* expression levels were investigated in mice testes at different postnatal days. The real-time PCR results showed that *Qrich2* began to be expressed obviously at postnatal day 15, reached the peak of expression at postnatal day 22, and then maintained a stable expression level afterward (Supplementary Fig. 2d). Thus, *Qrich2* is stably, highly and exclusively expressed in adult mice testes during evolution.

**Fig. 3:**
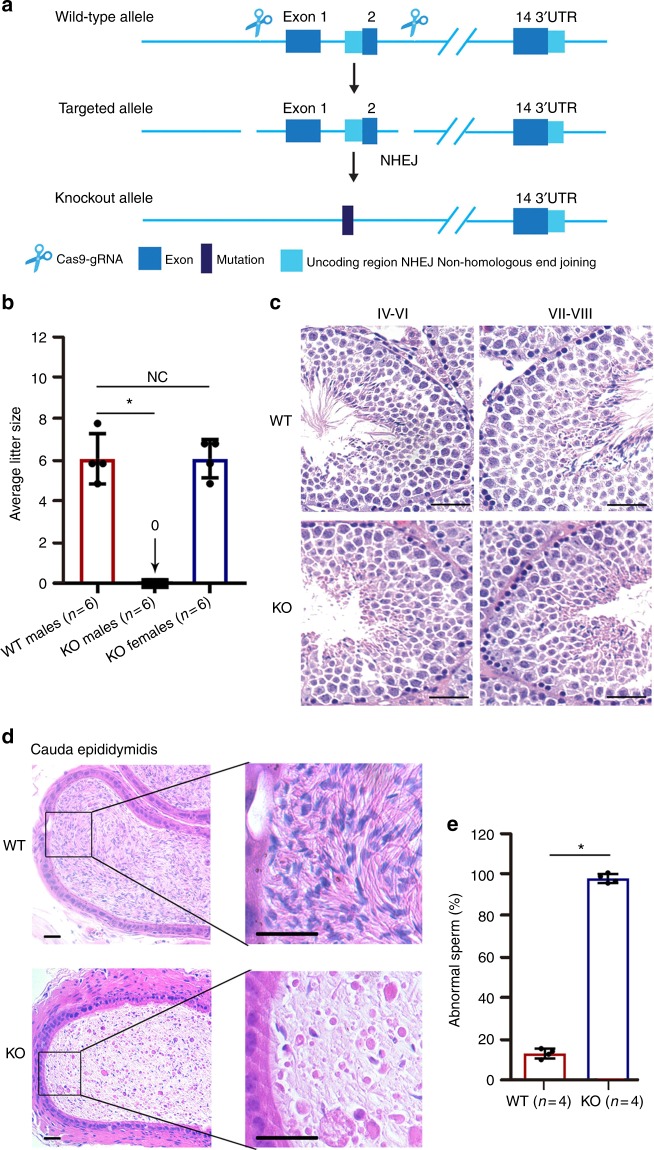
Deficiency of Qrich2 leads to MMAF in mice. **a** The schematic illustration of the targeting strategy for generating *Qrich2*-knockout mice. A frameshift mutation was generated by deleting exon 1 and exon 2 of *Qrich2*. **b** Fertility of wild-type (WT), knock out (KO) males and knock out (KO) females. KO male mice and KO female mice were bred with the WT mice and the average litter size was measured. KO males were completely sterile. (Student’s *t*-test; ^*^p < 0.05; NS, not significant; error bars, s.e.m) **c** The development of sperm flagella in stages IV-VIII of spermatogenesis in mice testes by H&E staining (scale bars, 20 µm). Lack of elongated flagella was observed in the spermatozoa of the KO mice compared with the WT mice. **d** The dysplasia of the sperm flagella and decreased sperm quantity were observed in the cauda epididymidis of the KO mice (scale bars, 10 µm). **e** The number of abnormal sperm in WT males and KO males. Almost 100% of sperm in KO males displayed abnormal flagella. (Student’s *t*-test; ^*^p < 0.05; error bars, s.e.m)

Considering that Qrich2 protein in mice is highly homologous to human QRICH2 protein and primarily expressed in mice testes, we established *Qrich2*-KO mice using the CRISPR-Cas9 technology to define the physiological role of QRICH2 in sperm flagellar formation. A frameshift mutation was generated by deleting exon 1 and exon 2 of *Qrich2* to achieve the knock out (Fig. 3a). We performed PCR, real-time PCR and western blotting to confirm the success of the *Qrich2*-KO mice (Supplementary Fig. 4a–c). After determining the deficiency of *Qrich2* in the KO mice, we first investigated the fertility of the KO mice. The KO male mice and KO female mice were bred with the WT mice for 5 months. After the mating period, we observed that the KO male mice were infertile, whereas the KO females had normal fertility (Fig. 3b).

**Fig. 4:**
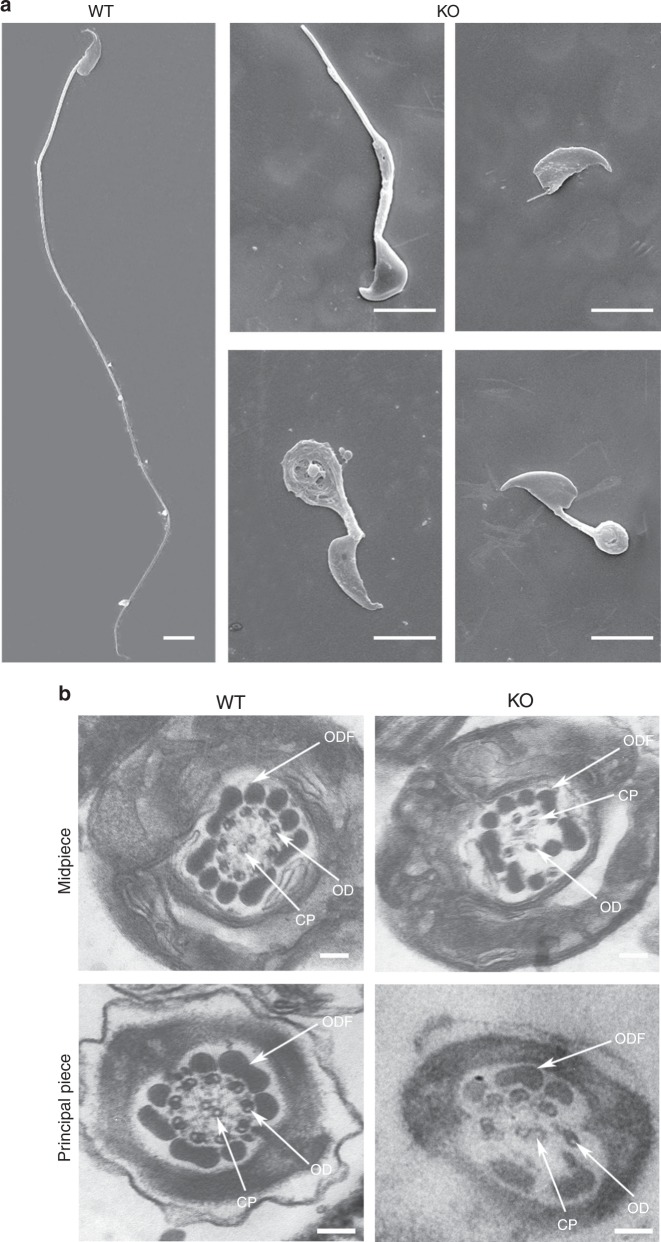
Electron microscopy analysis shows the typical MMAF phenotypes in KO mice. **a** The MMAF phenotypes in *Qrich2*-deficient mice by SEM (scale bars, 5 µm). The WT mice showed a normal flagellum, whereas the KO mice had short, coiled, or absent flagella. **b** numerous defects in the sperm flagellar ultrastructures in the KO mice. The cross-section of flagella from KO mice presents disorganized arrangement of CP and ODF, and incomplete OD in the mid-piece. And the absence of OD and ODF were observed in the principal piece. (scale bars, 150 nm),

Although no significant differences in testis size and weight were observed between the WT and KO mice, differences in epididymis size and weight were obvious (Supplementary Fig. 4d,e). To determine which spermatogenesis stage that Qrich2 affects on, we compared the histology of testicular tissue sections between the KO and WT mice by hematoxylin-eosin (H&E) staining. Flagellar formation defects were observed at stages VI-VIII in the KO mice compared with WT mice (Fig. 3c), which indicates that Qrich2 is involved at the stage of sperm flagellar formation. On analyzing the epididymis sections, few spermatozoa were observed, the flagella of which were aberrant in the KO mice (Fig. 3d). Light microscopy first demonstrated the morphological abnormalities of most sperm flagella in the KO mice (Fig. 3e, Supplementary Fig. 4f). The detailed and clear MMAF phenotypes in the KO mice were revealed by SEM (Fig. 4a). The sperm flagella of KO mice presented with short, coiled, bent, or other irregular shapes, which were consistent with the clinical phenotypes in the two subjects with MMAF. The ultrastructures of the sperm flagella were analyzed by TEM (Fig. 4b): in the mid-piece of most flagella of the KO mice, displacement of the CP and ODF and absence and/or incorrect localization of the OD were observed; the principal piece in the numerous sperm tails demonstrated missing of the ODF and abnormal 9 + 2 structure, for example, the 6 + 2 structure. All the defects of sperm flagella observed in the *Qrich2*-deficient mice strongly support the vital role of QRICH2 in the development of sperm flagella.

In addition, the computer-assisted sperm analysis (CASA) was used to exhaustively examine the sperm quality of the KO mice. The CASA results revealed reduced sperm motility, abnormal sperm flagellar morphology and decreased sperm count in the KO mice compared to the WT mice. Additional parameters showed a significant reduction in sperm locomotion in the KO mice. The detailed results are presented in Table 2. The diminished sperm movement in the KO mice compared to WT mice is directly reflected in Supplementary Movies 4 and 5. These data together indicate that *QRICH2* is involved in sperm flagellar formation and that its deficiency can result in MMAF and further cause male infertility.

**Table 2:** Semen analysis in wild-type (WT) and knock-out (KO) male mice by CASA

	Sample	
	WT	KO
Semen parameters		
Sperm concentration (10^6^/ml)^a,b^	26.59 ± 3.56	6.61 ± 1.35
Motility (%)^b^	73.36 ± 6.22	5.85 ± 3.00
Progressive motility (%)^b^	72.07 ± 6.44	3.60 ± 1.29
Sperm locomotion parameters		
Curvilinear velocity (VCL) (μm/s)^b^	74.88 ± 7.87	11.60 ± 1.71
Straight-line velocity (VSL) (μm/s)^b^	15.80 ± 3.60	0.61 ± 0.49
Average path velocity (VAP) (μm/s)^b^	31.27 ± 4.55	2.28 ± 0.30
Amplitude of lateral head displacement (ALH) (μm)^b^	1.04 ± 0.13	0.22 ± 0.01
Linearity (LIN)^b^	0.21 ± 0.04	0.06 ± 0.05
Wobble (WOB, = VAP/VCL)^b^	0.42 ± 0.05	0.20 ± 0.05
Straightness (STR, = VSL/VAP)^b^	0.50 ± 0.05	0.25 ± 0.17
Beat-cross frequency (BCF) (Hz)^b^	7.57 ± 1.14	0.75 ± 0.27

^a^Epididymides and vas deferens

^b^A significant difference *P* < 0.05 (*n* = 4 biologically independent WT mice or KO mice), Student’s *t*-test

### QRICH2 regulates flagellum-development-related proteins

**Fig. 5:**
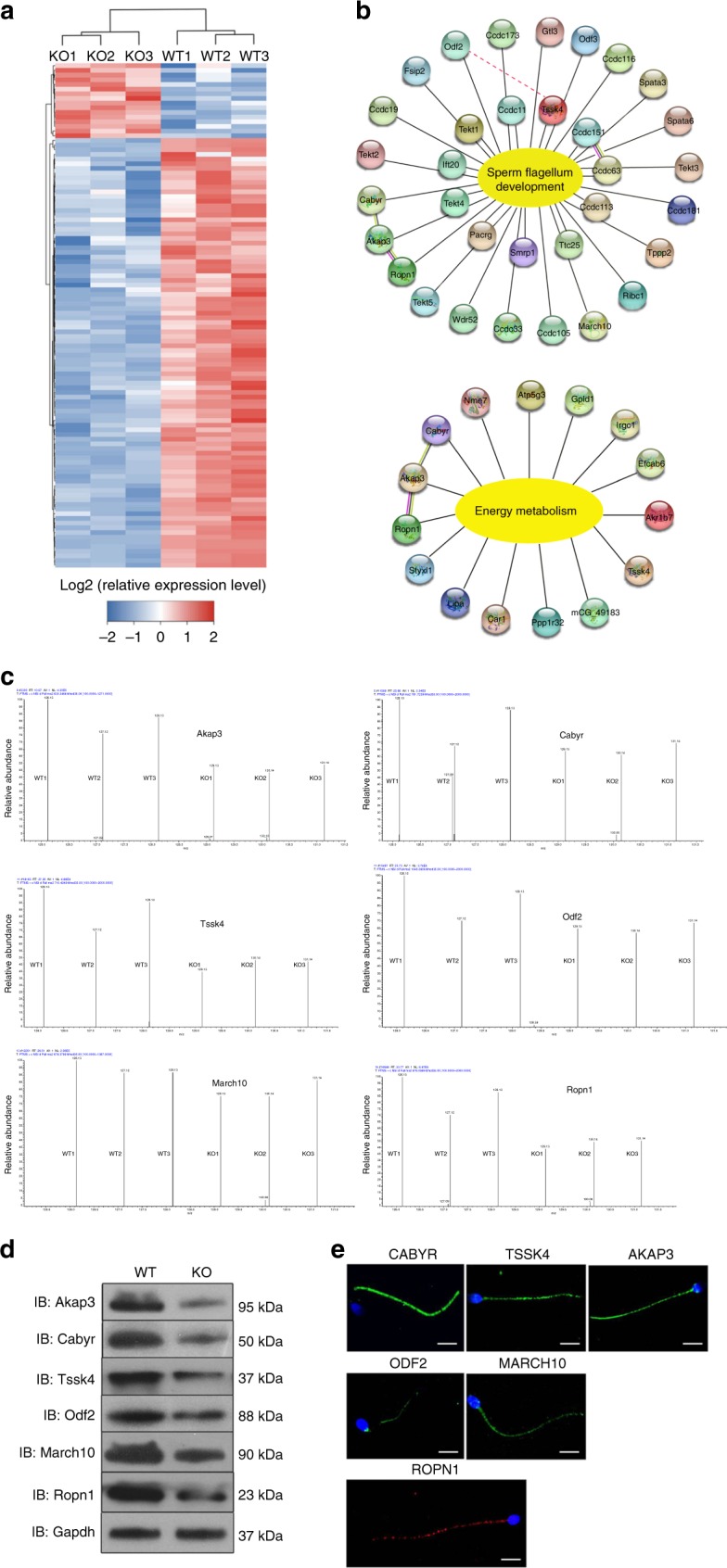
Proteins for sperm flagellum development are down-regulated in KO mice. **a** The heat map from three independent proteomic analyses of testes from the WT and KO mice. **b** The proteins down-regulated in the KO mice are associated with sperm flagellum development and related energy metabolism. **c** The different MS/MS spectra of Akap3, Odf2, Tssk4, March10, Ropn1, and Cabyr in the KO mice and the WT mice. **d** The expression of the key proteins in the testes of the WT and KO mice. The western blotting results confirmed the decreased expression of Akap3, Odf2, Tssk4, March10, Ropn1 and Cabyr in the KO mice compared with the WT mice. (*n* = 4 biologically independent WT mice or KO mice). **e** The location of the vital proteins in human spermatozoa by immunofluorescence staining. AKAP3 (green), ODF2 (green), TSSK4 (green), MARCH10 (green), CABYR (green), and ROPN1 (red) are dominantly expressed in human spermatozoa flagella. (blue, DAPI; scale bars, 5 µm)

To determine the molecular mechanism of QRICH2 during sperm flagellar formation, the protein expression levels in the testes of the WT and KO mice were investigated by a proteomics approach. A total of 6849 proteins were quantified, of which 108 proteins were identified to be expressed differentially, including 92 down-regulated proteins and 19 up-regulated proteins (Fig. 5a). Among the decreased proteins, 33 are involved in the development of sperm flagella, and 15 are required for energy metabolism, which is crucial for sperm motility^24–26^ (Fig. 5b). According to the literature, six of these proteins, namely, A-kinase anchoring protein (AKAP3), outer dense fiber of sperm tails 2 (ODF2), testis specific serine kinase 4 (TSSK4), calcium binding tyrosine phosphorylation regulated (CABYR), rhophilin associated tail protein 1 (ROPN1) and membrane associated ring-CH-type finger 10 (MARCH10), were directly related to the formation of the sperm tail; the complete ablation of these proteins caused impaired development of sperm flagella in mouse models^10–15^. Therefore, we focus on these six proteins. Western blotting results further confirmed the decreased expression of these six proteins in the KO mice compared to the WT mice, which was consistent with TMT quantification results (Fig. 5c, d). Meanwhile, the bindings between QRICH2 and AKAP3, ODF2 or TSSK4 were confirmed by exogenous Co-IP in HEK 293 cells (Supplementary Fig. 5a). Furthermore, these proteins, similar to QRICH2, were predominantly localized in the human spermatozoa flagella, supporting their vital roles in sperm tail formation (Fig. 5f).

To confirm the decreased expression of Akap3, Tssk4, Ropn1, March10, Cabyr, and Odf2 was caused by the deficiency of Qrich2 directly, but not by the sperm tail defects, we performed further research on heterozygous (Ht) *QRICH2* mice which have the normal flagella development (Supplementary Fig. 5b). The western blotting results showed that the expression of these six proteins in Ht mice is lower than in WT mice (Supplementary Fig. 5c). In addition, when we knocked down *QRICH2* in NT2 cells (human testicular embryonic carcinoma cell) which have been identified to express QRICH2, AKAP3, CABYR, ODF2, and TSSK4, and the decreased protein amount of AKAP3, CABYR, ODF2, and TSSK4 was observed in these knocked-down cells compared to the control cells (Supplementary Fig. 5d), Therefore, the decreased levels of the proteins which we focus on are caused by the reduced expression of QRICH2 directly but not the lack of sperm tail structures. Taken together, *QRICH2* is involved in sperm tail formation through regulating the expression of sperm tail structural proteins and energy metabolism proteins.

**Fig. 6:**
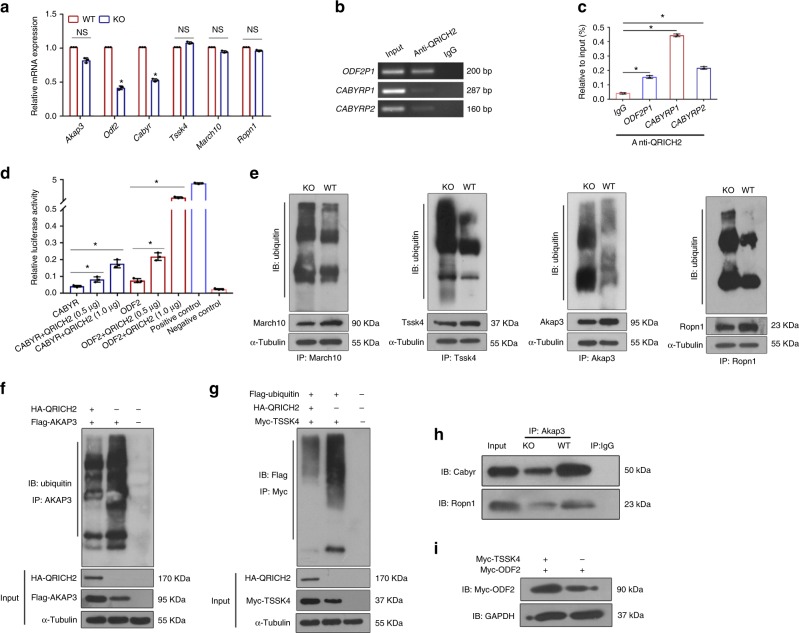
The mechanisms of QRICH2 regulating sperm flagellar formation. **a** The mRNA levels of *Akap3*, *Odf2*, *Tssk4*, *March10*, *Cabyr* and *Ropn1* in the testes of the KO and WT mice. The reduced mRNA levels of *Odf2* and *Cabyr* in the KO mice were detected by real-time PCR (Student’s *t*-test; *n* = 3 independent experiments; ^*^p < 0.05; NS, not significant; error bars, s.e.m). **b** One QRICH2-occupied site in the *ODF2* promoter and two QRICH2-occupied sites in the *CABYR* promoter were detected by ChIP-PCR assays of human testicular tissues. Input DNA and normal mouse IgG pulldowns were used as positive control and negative control, respectively. **c** ChIP-qPCR results showed the increased binding of QRICH2 to the *ODF2* and *CABYR* promoter regions in the group with QRICH2 antibody compared to that with IgG (Student’s *t*-test; *n* = 3 independent experiments; ^*^p < 0.05; error bars, s.e.m). **d** QRICH2 could enhance the transcriptional activity of *ODF2* and *CABYR* by luciferase reporter assays (Student’s *t*-test; *n* = 3 independent experiments; ^*^p < 0.05; error bars, s.e.m). **e** The increased ubiquitination levels of Akap3, Tssk4, Ropn1 and March10 in the testes of the KO mice compared with that in the WT mice. **f**, **g** QRICH2 decreased the ubiquitylation of exogenous AKAP3 (**f**) and TSSK4 (**g**) in HEK 293 cells. The cells were harvested after a 6-h treatment with 10 μM of MG132. **h** The binding of Ropn1 and Cabyr to Akap3 was weakened in the testes of the KO mice compared with that in the WT mice. **i** Overexpression of TSSK4 could increase the protein levels of ODF2

Real-time PCR was used to investigate whether Qrich2 regulated these proteins via their transcriptional levels. We observed that the mRNA levels of *Odf2* and *Cabyr* from KO-mice testes were approximately 60 and 52% lower than that of WT mice, respectively (Fig. 6a). Meanwhile, the significant decreased mRNA levels of *Cabyr/CABRY* and *Odf2/ODF2* were also observed in Ht mice or QRICH2-knocked-down NT2 cells, compared with WT mice and control cells, respectively (Supplementary Fig. 5e,f). To find out whether QRICH2 acted as a trans-acting factor to enhance the transcriptional activity of *ODF2* and *CABYR*, chromatin immunoprecipitation (ChIP)-PCR was performed and the results showed that QRICH2 could bind to the promoter regions of *CABYR* and *ODF2* (Fig. 6b). Similarly, the ChIP-qPCR results showed the enhanced binding of QRICH2 to the promoter fragments of *ODF2* and *CABYR* approximately 4-fold, 11-fold, and 5-fold respectively, compared with the negative control (Fig. 6c, Supplementary Fig. 5g). Furthermore, we constructed luciferase reporter vectors containing *ODF2* and *CABYR* promoter fragments and co-transfected them with QRICH2 expression vector individually. The luciferase activity of the constructs with the promoter fragments of *ODF2* and *CABYR* were increased in gradient when QRICH2 was overexpressed in gradient in HEK 293 cells (Fig. 6d). These results indicate that QRICH2 enhances *ODF2* and *CABYR* gene expression by acting as a trans-acting factor.

The limited difference in the mRNA levels of *Akap3*, *Tssk4*, *Ropn1*, and *March10* between the KO and WT mice indicates that Qrich2 may not affect their transcription (Fig. 6a). Accordingly, we hypothesized that QRICH2 may be involved in the post-transcriptional regulation of these four proteins. The degradation levels of these proteins were determined by analyzing the ubiquitination levels of Akap3, Tssk4, Ropn1, and March10 between the KO and WT mice, which is the most common mechanism for protein degradation. As expected, the increased ubiquitination degradation of Akap3, Tssk4, Ropn1, and March10 was observed in the KO mice compared with the WT mice (Fig. 6e). We further confirmed the influence of QRICH2 on AKAP3 and TSSK4 by inhibiting their ubiquitination degradation in vitro. We observed that the overexpression of QRICH2 significantly inhibited the ubiquitination of exogenous AKAP3 and TSSK4 in the HEK 293 cells (Fig. 6f, g). These data suggest that QRICH2 is a key molecule that regulates sperm flagellar formation by stabilizing the related proteins through suppressing their ubiquitination degradation.

**Fig. 7:**
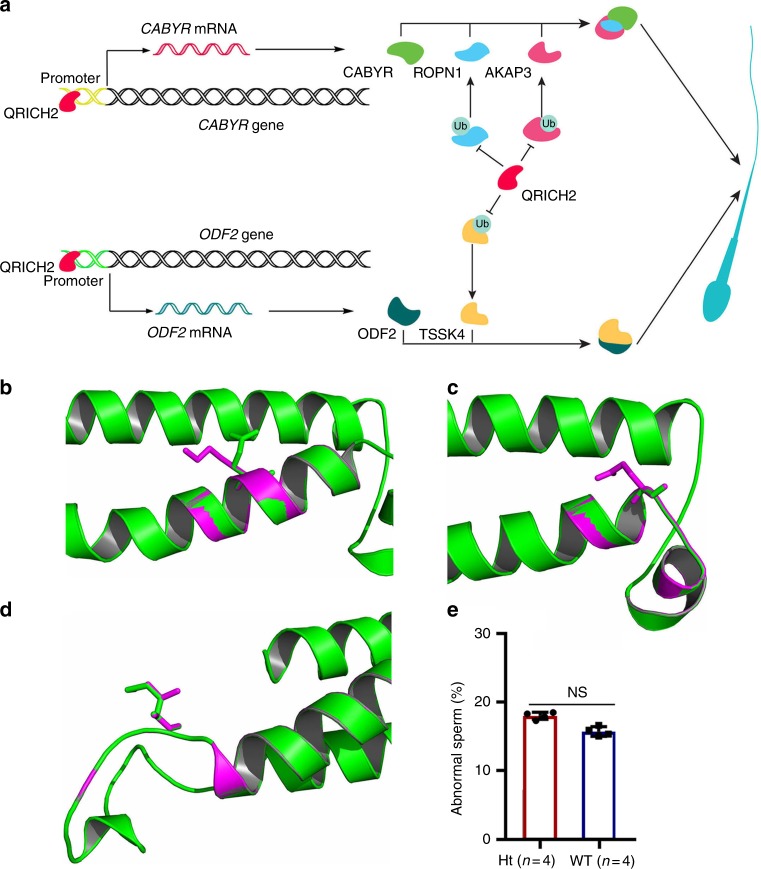
The mechanisms were elucidated by proposed models. **a** The mechanisms of QRICH2 modulating the development of sperm flagella. QRICH2 promoted sperm flagellar formation through upregulating ODF2 and CABYR expression as a transcription factor, inhibiting ubiquitin-mediated degradation of AKAP3, TSSK4, and ROPN1 and finally reinforcing the interaction of AKAP3/CABYR/ROPN1 and TSSK4/ODF2 during the biogenesis of sperm flagella. **b**–**d** Effects of *QRICH2* mutations on the structural conformation of QRICH2 protein. Superimposed structures of wild type (green) and mutant (purple) of QRICH2. The structures of QRICH2 protein were changed by the mutations of c.3313C>A [p.Q1105K] (**b**), c.3335G>A [p.G1112E] (**c**) and c.4039A>G [p.M1347V] (**d**). **e** The difference of the abnormal sperm tail number between WT mice and Ht mice was not significant. (Student’s *t*-test; *n* = 3 biologically independent WT mice or Ht mice; NS not significant; error bars, s.e.m)

It has been suggested that ROPN1 and CABYR utilize docking interactions with AKAP3 to regulate cilia and flagellar formation through the PKA-related pathway^27–29^. In this study, we observed that the binding of Ropn1 and Cabyr to Akap3 was weakened in the KO mice compared with the WT mice by Co-IP assay (Fig. 6h). Furthermore, TSSK4 was reported to influence sperm flagellar formation by enhancing the phosphorylation state of ODF2, which is a prominent protein of the sperm tail outer dense fibers^13,30–33^. Because no commercial antibody was available, we did not confirm that TSSK4 can increase the phosphorylation of ODF2. However, we found that the overexpression of TSSK4 could increase ODF2 expression (Fig. 6i). In all, QRICH2 could suppress the ubiquitination degradation of AKAP3 and ROPN1 and facilitate CABYR expression as a trans-acting factor, correspondingly reinforcing the binding of AKAP3, ROPN1, and CABYR in sperm flagella. Additionally, QRICH2 regulates the formation of outer dense fiber in sperm flagella by directly enhancing the transcription of ODF2 and stabilizing TSSK4 through inhibition of its ubiquitination degradation, which is a crucial factor for ODF2 phosphorylation. Overall, these findings reveal that QRICH2 is required for maintaining the normal structure of sperm flagella by regulating key proteins in the related signaling pathways (Fig. 7a).

### *QRICH2* mutations detected in patients with asthenozoospermia

Two homozygous nonsense mutations of *QRICH2* were observed in two MMAF patients from two consanguineous families. Additionally, *Qrich2*-deficient male mice showed MMAF phenotypes and infertility. These results suggested that *QRICH2* plays a crucial role in male reproduction associated with MMAF. The results of proteomics analysis showed that several proteins which are not only related to the development of sperm tail structure, but also to energy metabolism were significantly reduced in *Qrich2*-deficient mice. The energy metabolism in sperm flagella provides the sliding force for flagella beating which directly affects the sperm motility. Reduced sperm motility is the basic character for asthenospermia which is defined as a semen sample containing less than 40% of motile sperm and less than 32% with progressive motility^34^. The expressions of ROPN1 and CABYR have been observed remarkably decreasing in asthenospermia subjects in comparison with the conrols^35^. Furthermore, the mutations of dynein genes were found in isolated non-syndromic asthenozoospermia patients^36^. Consequently, we suppose that whether *QRICH2* mutations are associated with asthenospermia. To investigate the variation of *QRICH2* in interfile males with asthenospermia, we sequenced all 18 exons and their flanking intronic regions of *QRICH2* in 150 asthenospermia patients. Finally, six heterozygous mutations, including five missense mutations and one nonsense mutation, were observed in six subjects (Table 3). These mutations were predicted to be pathogenic by bioinformatic tools: SIFT^37^, PolyPhen-2^38^, and M-CAP^39^ (Table 3). To further understand the effect of these mutations, we investigated the conformational changes of QRICH2 protein caused by these mutations with SWISS-MODEL^40^ (https://swissmodel.expasy.org/) and PyMoL software^41^ (1.3r1, DeLano Scientific LLC). The results of silico analysis showed that the mutations of c.3313C>A [p.Q1105K], c.3335G>A [p.G1112E], and c.4039A>G [p.M1347V] could change the conformation of QRICH2 protein (Fig. 7b, d). Because no gap-free homology templates for the spacer fragments (aa 550–600 and aa 1300–1360) could be identified in the PDB database, the effects of c.1706T>A [p.V569E] and c.4039A>G [p.M1347V] on QRICH2 protein structure were not predicted.

**Table 3:** Analysis of *QRICH2* mutations in asthenozoospermia patients

	Human subject					
	P76	P19	P72	P34	P364	P241
cDNA mutation^a^	c.3380T>A	c.1706T>A	c.3313C>A	c.3335G>A	c.4039A>G	c.4481G>A
Protein alteration	p.L1127^a^	p.V569E	p.Q1105K	p.G1112E	p.M1347V	p.R1494H
Mutation type	Nonsense	Missense	Missense	Missense	Missense	Missense
Allele frequency in ExAC Browser	0	0	0	0.000008237	0.00001648	0.0009482
Function prediction						
SIFT	NA	Deleterious	Deleterious	Deleterious	Deleterious	Deleterious
PolyPhen-2	NA	Probably damaging	Probably damaging	Probably damaging	Probably damaging	Probably damaging
M-CAP	NA	Possibly pathogenic	Possibly pathogenic	Possibly pathogenic	Possibly pathogenic	Possibly pathogenic

*NA* not available

^a^The accession number for *QRICH2* is GenBank: NM_032134.2

Accordingly, no homozygous mutations or compound heterozygous mutations were found in the asthenospermia patients, suggesting that the recessive *QRICH2* may just relate to MMAF. Several variants were indeed detected in the normal controls (200 normal fertility males) (Supplementary Table 1), whereas those rare and deleterious heterozygous mutations of *QRICH2* were found only in asthenospermia (asthenozoospermia: 6/150; control: 0/200; *p* < 0.006, the Fisher exact test, two-sided), indicating that deleterious heterozygous mutations of *QRICH2* may increase the risk of asthenospermia. To further prove the vital role of heterozygous mutations of *QRICH2* in sperm movement, we performed research on epididymal spermatozoa of Ht mice. We observed that the morphology of most sperm is normal in Ht mice (Supplementary Fig. 5b) and the number of abnormal sperm tail was not significantly different between Ht mice and WT mice (Fig. 7e). Notably, by CASA, the sperm motility parameters were decreased in Ht mice compared with WT mice (Supplementary Table 2). The diminished sperm movement in the Ht mice compared to WT mice is directly showed in Supplementary Movies 6 and 7. Given that the reduced sperm motility in Ht mice closely resembles the sperm phenotype of human asthenospermia, these data suggest that the deleterious heterozygous mutations of human *QRICH2* may contribute to asthenozoospermia.

## Discussion

Impaired sperm motility is a major contributor to male infertility, and the sperm flagellum is the key factor affecting sperm motility. Variations in the genes involved in the formation of sperm flagella can cause abnormalities in sperm flagellum morphology and further reduce sperm motility, thus impairing male fertility. Several genes have been reported to be associated with the development of the sperm flagellum in mouse models, whereas mutations in these genes in mice are rarely identified with those in humans with MMAF, except *AKAP4, CCDC39, DNAH1, CFAP43*, *CFAP44*, and *CFAP69* (refs. ^4,5,8,16–19^). Therefore, it is crucial to explore the pathogenic genes of MMAF in humans and further understand the mechanisms required for the structure of sperm tails and their effect on fertility.

In this study, we detected two homozygous nonsense mutations of the *QRICH2* in two MMAF patients from two consanguineous families. It is the first time that *QRICH2* has been identified to cause MMAF in humans, providing a new genetic diagnostic cue for male infertility. Our further investigations suggest that the two variants result in dysplasia of sperm flagellum: almost all observed sperm from affected patients exhibited multiple morphological abnormalities of the sperm flagella. Additionally, irregular sperm ultrastructures were observed by TEM: peripheral fibers or microtubules were absent, such as in 9 + 0 and 6 + 0 axonemal arrangement. Furthermore, the *Qrich2*-deficient mouse models showed MMAF phenotypes and abnormal flagellum ultrastructure, supporting the vital role of *QRICH2* in the development of the sperm flagellum and that homozygous loss-of-function mutations in *QRICH2* cause the abnormal development of the sperm flagellum, which leads to male infertility.

*QRICH2* is evolutionarily conserved in several species, indicating its fundamental role in organisms. However, the functions of *QRICH2* have not been characterized so far. QRICH2 contains three principal domains: Glutenin hmw super family, SMC_N super family and DUF4795 (https://www.ncbi.nlm.nih.gov). The SMC_N super family is the only known functional domain in QIRCH2. The structural maintenance of chromosomes (SMC) super family proteins have ATP-binding domains at the N-termini and C-termini, which are involved in chromatin and DNA dynamics^42–44^. SMC-like domains are highly conserved in some ciliary and centrosomal proteins, which are related to intraflagellar transport^45–48^, suggesting that SMC-like domains might represent signature sequences that are recognized during these transport processes. Importantly, the mutations in the SMC_N domain of *CFAP43* and *CCDC39* have been reported to be associated with MMAF^8,18^. Hence, QRICH2 containing the SMC_N domain supports that QRICH2 is required for sperm flagellar development.

*QRICH1* is the homologous gene of *QRICH2*, whose mutations are associated with developmental delay^49,50^. However, to date, there are no studies reporting the functions of *QRICH2*. To understand the molecular mechanism of QRICH2 in the development of the sperm flagella, we used a proteomics approach to compare the different expression of proteins in the testes of the WT and KO males. Intriguingly, 33 proteins suggested to be associated with the formation of sperm tail were significantly decreased in the KO mice compared with the WT mice^9,51–54^. Among them, *CFAP44* variants have been reported to cause MMAF in humans^8,16^. Mice missing some of these genes have demonstrated abnormal sperm tail structures (*Spata6, Tekt4, Odf2, Ropn1*, and *Cabyr*)^10–15^. A potential mechanism is that ROPN1 and CABYR are involved in the development of the sperm flagella though binding with AKAP3 and further regulating PKA signaling^28,29^. In this study, we observed that QRICH2 inhibited the ubiquitination degradation of AKAP3 and ROPN1, increased CABYR mRNA levels, and further maintained the complex of AKAP3, to which ROPN1 and CABYR stably bind in the sperm flagella. Meanwhile, QRICH2 stabilized TSSK4/ODF2-mediated pathways, which are crucial for sperm tail structure growth^12,13,30–33^, by promoting their protein and mRNA expression. In addition, QRICH2 restrained the ubiquitination degradation of MARCH10, a microtubule-associated E3 ubiquitin ligase regulating the formation and maintenance of the flagella in developing spermatids. Our findings elucidate the potential mechanisms of QRICH2 in regulating the development of the sperm flagellum and provide additional cues for future research on the functions of QRICH2.

In this study, no homozygous mutations but six deleterious heterozygous variants were detected in patients with asthenozoospermia. Remarkably, in addition to the deleterious heterozygous variant (c.3313C>A), P72 carried a rare SNP (rs780426606). For P241, two other SNPs (rs67948583 and rs73999020) with extremely low frequency were determined. We therefore speculate that the compound deleterious heterozygous variants and rare SNPs of *QRICH2* may be the risk for P72 and P241. Besides that, the Ht mice represented the decreased sperm motility. Thus so, the pathologic heterozygous variants of *QRICH2* could increase the risk for asthenozoospermia. Certainly, this theory needs to be further investigated.

In summary, our genetic and functional data based on human subjects and mouse models strongly suggest that homozygous loss-of-function mutations of *QRICH2* are a novel genetic cause of MMAF. Additionally, the underlying mechanism that QRICH2 regulates sperm flagellar development was elucidated in this study. The screening of the deleterious mutations of *QRICH2* could be important for clinical molecular diagnosis of male infertility. As the findings about *QRICH2* in this study are novel, further research is required on this unfamiliar gene to explore the importance of *QRICH2* in male fertility.

## Methods

### Study participants

Two interfile males with MMAF from two consanguineous families were enrolled at the Human Sperm Bank of West China Second University Hospital of Sichuan University and the Center of Reproductive Medicine at the First Affiliated Hospital of Nanjing Medical University. Family members of these patients were additionally recruited. Furthermore, 150 patients of Han ethnicity with primary infertility and diagnosed with asthenozoospermia (the percentage of progressively motile sperm < 32%) from the Department of Medical Genetics, the Department of Urology in West China Hospital, Sichuan University were enrolled for the population study. Patients were excluded any significant androgenic or endocrine abnormalities in a physical examination, hormone analysis and scrotal ultrasound, including hypogonadotropic hypogonadism, cryptorchidism, varicocele, seminal ductal obstruction, testicular trauma and tumor. The patients have chromosomal aberration were also excluded. The detailed sperm parameters of the patients were listed in Supplementary Data 2. A total of 200 unrelated Han Chinese males with normal fertility (fathering at least one offspring by natural fertilization) and sperm quality (sperm concentration ≥ 15 million/ml; the percentage of progressively motile sperm ≥ 32%) were recruited from volunteers served as the control group for the population study. The inclusion and exclusion criteria used in this study were selected according to previously published reports and the guidelines of the World Health Organization^4–6,34,55^. The study was approved by the Ethical Review Board of West China Second University Hospital, Sichuan University. Informed consent was obtained from each subject in our study.

### Whole-exome sequencing (WES) and Sanger sequencing

Genomic DNA was isolated from peripheral-blood samples of the subjects using a whole blood DNA purification kit (QIAGEN). Whole-exome sequencing (WES) was performed on the two MMAF patients from two consanguineous families. 1 μg of genomic DNA was utilized for exon capture using the Agilent SureSelect Human All Exon V6 Kit and sequenced on the Illumina HiSeq X system according to the manufacturer’s instructions. The average sequencing depth on target was 108.11, and the ratio of target fraction covered at minimum 10× was 99.4%.

Reads were mapped to the reference genome (UCSC hg19) by the Burrows-Wheeler Aligner (BWA) software to get the original mapping result in BAM format, and duplicates were marked and removed using Picard (http://broadinstitute.github.io/picard/index.html). ANNOVAR was performed for functional annotation through a variety of databases, such as dbSNP, 1000 Genomes Project, ExAC and HGMD. After filtering, the retained nonsynonymous SNVs were submitted to PolyPhen-2, SIFT, MutationTaster and CADD for functional prediction.

Candidate pathogenic variants in the family members and 18 coding exons and their flanking intronic regions of *QRICH2* in the unrelated population were validated by Sanger sequencing. PCR amplification was performed with Dyad Polymerase (Bio-Rad Laboratories). DNA sequencing of PCR products was conducted on an ABI377A DNA sequencer (Applied Biosystems). The primers for PCR are listed in Supplementary Table 3.

### Tandem Mass Tagging (TMT) proteomics analysis

The primary experimental procedures for TMT proteomics analysis include protein preparation, trypsin digestion, TMT labeling, HPLC fractionation, LC-MS/MS analysis and data analysis. The detailed procedure is presented in the Supplementary methods. The TMT proteomics analysis in our research is supported by Jingjie PTM BioLabs.

### Generation of *Qrich2*-deficient mouse models

All animal experiments were performed in accordance with the recommendation of the Guide for the Care and Use of Laboratory Animals of the National Institutes of Health. The animal experiments were approved by the Experimental Animal Management and Ethics Committee of West China Second University Hospital, and all animal works were performed in accordance with the guidelines and regulations the Committee. The frameshift mutation was generated using the CRISPR-Cas9 technology by the non-homolog recombination method. The guide RNAs were designed against the 5′UTR and intron 2 of *Qrich2*. The Cas9 mRNA and gRNA were obtained by in vitro transcription assay and then microinjected into the cytoplasm of single-cell C57BL/6J embryos. The injected embryos were transferred into the oviducts of pseudopregnant mice. The frameshift mutation in *Qrich2* was identified in the founder mice and their offspring by PCR, real-time PCR and western blotting. The primers for PCR and real-time PCR are listed in Supplementary Table 3 and Table 4.

### Plasmid construction

The full-length cDNA of *AKAP3*, *TSSK4*, *ODF2*, and *QRICH2* was synthesized and separately cloned into pENTER (Vigene), pCMV6-Entry (Origene), pCMV6-Entry, and pcDNA^TM^3.1^(+)^ (Invitrogen) vectors containing a FLAG, Myc, and HA tag sequence. *CABYR* and *ODF2* promoter fragments were amplified and cloned into the pGL3-Basic luciferase reporter vector (Promega). The shRNAs designed to interfere with QRICH2 expression and the negative control SuperSilencing shRNA (shNC) were synthesized and cloned into the psi-LVRU6GP vector by GeneCopoeia. The target sequences of QRICH2 shRNAs were 5′-GGGTTCACTTCCTTAACATCA-3′. The primers for promoter region amplification are listed in Supplementary Table 5.

### Real-time PCR

Total RNA of mice tissues was extracted using TRIzol reagent (Invitrogen) and was converted to cDNA using a RevertAid First-Strand cDNA Synthesis Kit (ThermoFisher). Real-time PCR was performed using SYBR Premix Ex Taq II (TaKaRa) on an iCycler RT-PCR Detection System (Bio-Rad Laboratories). The ΔΔCT method was used for data analysis^56^. Each assay was performed in triplicate for each sample. The *Gapdh* gene was used as an internal control. The primers for real-time PCR are listed in Supplementary Table 4.

### Dual-luciferase reporter assays

Two plasmids, including the pGL3-ODF2 promoter region and pGL3-CABYR promoter region, were separately co-transfected with the HA-QRICH2 plasmid and individually transfected into HEK 293 cells, with the pGL3-control plasmid as the positive control and the pGL3-basic plasmid as the negative control. After 48 h, the luciferase activity of the cell lysates was analyzed using a dual-luciferase reporter assay system (Promega).

### Antibodies

The antibodies used in western blotting, Co-IP and immunofluorescence staining are as follows: anti-QRICH2 (1:200, sc-514279, Santa Cruz Biotechnology), anti-QRICH2 (1:50, HPA052219, Sigma-Aldrich) for immunofluorescence staining of mice sample, anti-QRICH2 (1:50, HPA021935, Sigma-Aldrich) for immunofluorescence staining of human sample, anti-AKAP3 (1:100, provided by Prof. Huayu Qi) for immunofluorescence staining, anti-AKAP3 (1:100, 13907-1-AP, Proteintech) for Co-IP, anti-ODF2 (1:500, 12058-1-AP, Proteintech), anti-CABYR (1:500, 12351-1-AP, Proteintech), anti-ROPN1 (1:200, sc-130455, Santa Cruz Biotechnology), MARCH10 (1:500, bs-10732R, Bioss), anti-TSSK4 (1:400, A7861, ABclonal), anti-ubiquitin (1:1000, ab7780, Abcam), anti-Flag Tag (1:500, 66008-2-Ig, Proteintech), anti-Myc Tag (1:1000, sc-764, Santa Cruz Biotechnology), anti-α-Tubulin (1:2000, T7451, Sigma-Aldrich) and anti-GAPDH (1:5000, ab8245, Abcam). Anti-AKAP3 mouse monoclonal antibody for western blotting was kindly provided by Prof. Huayu Qi. Anti-TSSK4 rabbit polyclonal antibody for Co-IP was kindly provided by Prof. Long Yu.

### Western blotting

The proteins of the cultured cells and mice testes tissues were extracted using a universal protein extraction lysis buffer (Bioteke) containing a protease inhibitor cocktail (Roche). The denatured proteins were separated on 10% SDS-polyacrylamide gels and transferred to a polyvinylidene difluoride (PVDF) membrane (Millipore) for the immunoblot analysis.

### Co-immunoprecipitation (Co-IP)

The extracted proteins were incubated with 3 µg of target antibodies overnight at 4 °C. Next, 50 μl of Protein A/G magnetic beads (Invitrogen) was added to each incubation sample for 1 h at room temperature. The beads were washed three times with 1× PBS. Finally, the co-immunoprecipitated proteins were eluted by standard 1× SDS sample buffer and heated for 10 min at 70 °C and then separated on 10% SDS-polyacrylamide gels and PVDF membranes for the immunoblot analysis.

### Chromatin immunoprecipitation (ChIP)-PCR and ChIP-qPCR

ChIP assays were performed using a ChIP-IT Express Enzymatic Kit (Active Motif). In brief, the testes tissues were fixed with 1% formaldehyde, and chromatin was extracted in cell lysis buffer and then in nuclear lysis buffer with protease inhibitor cocktail and then sheared by sonication (Bioruptor, Scientz) on ice to a mean length of 500 bp. 1% of the sheared chromatin was retained as a positive control input DNA and another portion was precipitated by incubation with IgG as a negative control in the subsequent PCRs. The remaining chromatin was precipitated by incubation with antibodies against QRICH2. PCR and qPCR were performed to amplify the target regions of *CABYR* and *ODF2* which QRICH2 binding to. The qPCR data were analyzed by Fold Enrichment Method^57^ and Percent Input Method^58^ simultaneously, and in Percent Input Method, the value of target DNA fragments that were enriched in testes tissue was normalized to the value of the 1% input DNA. Each assay was performed in triplicate to confirm the reproducibility of the results. The primers are listed in Supplementary Table 3.

### Flow cytometric analysis and cell sorting

The testes from 8-week-old male mice were removed and decapsulated. Firstly, the seminiferous tubules were incubated in 10 ml 1× PBS containing 90 mg/ml of collagenase (Invitrogen) with continuous agitation for 15 min at 32 °C and then allowed to sediment for 5 min and the supernatant was removed. Next, the pellet was resuspended in 10 ml of PBS with 60 mg/ml of trypsin (Sigma-Aldrich) and 1 µg/ml of DNase (Promega), and incubated under the same conditions for 15 min. After gently being pipetted with a Pasteur pipette, the cell suspension was centrifuged at 400 × *g* for 10 min and then washed three times with 1× PBS, filtered using 40 µm nylon mesh to remove cell clumps and last resuspended in HEPES-buffered RPMI containing 0.5% BSA. After the testicular single cell suspensions were obtained, two million cells were diluted in 2 ml of 1× PBS buffer and stained with Hoechst 33342 (5 μg/ml; Sigma) for 1 h at 32 °C. Before analysis, PI (2 μg/ml; Sigma) was added to exclude dead cells. Finally, cell analysis and sorting were performed on a FACScalibur flow cytometer (Beckman Coulter, CA, USA) equipped with a cell sorting system.

### Immunofluorescence staining

After being washed with Sperm Washing Medium (CooperSurgical Inc.), the spermatozoa samples or the selected germ cells were fixed onto slides with 4% paraformaldehyde, permeabilized with 0.5% Triton X-100, and blocked with 1% BSA. Next, the slides were sequentially incubated with primary antibodies overnight at 4 °C. The slides were washed in 1× PBS, incubated with Alexa Fluor 488 (1:1000, A21206, Thermo Fisher) or Alexa Fluor 594 (1:1000, A11005, Thermo Fisher) labeled secondary antibodies for 1 h at room temperature, and then counterstained with 4,6-diamidino-2-phenylindole (DAPI, Sigma-Aldrich) to label the nuclei. Images were acquired using a laser scanning confocal microscope (Olympus).

### Histology hematoxylin-eosin (H&E) staining

Testicular and epididymal tissue samples from male mice were fixed with 4% paraformaldehyde overnight, dehydrated in ethanol, embedded in paraffin, and sectioned at 5 μm. The sections were stained routinely with hematoxylin and eosin for histological examination.

### Electron microscopy

For scanning electron microscopy (SEM) assay, the spermatozoa samples were fixed onto slides of 1 cm diameter using 2.5% glutaraldehyde for 4 h at 4 °C. After washing the slides with 1× PBS three times and post-fixing in 1% osmic acid for 1 h at 4 °C, dehydration was performed using 30, 50, 75, 95, and 100% ethanol sequentially, and the slides were dried using a CO_2_ critical-point dryer (Eiko HCP-2, Hitachi). Finally, the slides were mounted on aluminum stubs, sputter-coated by an ionic sprayer meter (Eiko E-1020, Hitachi), and analyzed by SEM (Hitachi S3400) under an accelerating voltage of 10 kV.

For the transmission electron microscopy (TEM) assay, each semen sample was rinsed in Sperm Washing Medium. The semen samples were pre-fixed in 3% glutaraldehyde, post-fixed in 1% buffered OsO4, dehydrated through gradient acetone solutions, and embedded in Epon 812. Before ultrathin-sectioning, a half-thin section was made to enable sperm location under a light microscope. The ultrathin sections were double-stained with both lead citrate and uranyl acetate and then analyzed under a TEM (TECNAI G2 F20, Philips) with an accelerating voltage of 80 kV.

### STORM imaging

Sperm cells were fixed and immunostained with rabbit anti-QRICH2 (1:50, HPA021935, Sigma-Aldrich) and mouse anti-α-Tubulin (1:100, T7451, Sigma-Aldrich) overnight at 4 °C. After washed in 1× PBS, sperm cells were incubated with Atto 488 (1:500, 41051, Sigma-Aldrich) or Alexa fluor 568 (1:500, A10037, Thermo Fisher) secondary antibodies for 1 h at room temperature. Finally, the sperm samples were stained with imaging buffer (PBS with the addition of 100 mM mercaptoethylamine at pH 8.5, 5% glucose (w/v), 0.5 mg/ml glucose oxidase (Sigma-Aldrich), and 40 μg/ml catalase (Roche Applied Science)) and illuminated at 657 nm for imaging. The 501 nm and 568 nm lasers were used to activated Atto 488, and Alexa fluor 568, respectively. A cylindrical lens (focal length = 1 m) used for 3D localization was inserted into the detection path to enable determination of z positions from the ellipticities of the molecular images and the *x* and *y* positions from the centroid positions.

### Statistical analysis

The statistical analyses were performed using the SPSS 17.0 software (IBM Company). Student’s *t*-test and Fisher exact test were used to compare the observed indexes between the experimental groups. The *P* values less than 0.05 were considered significant.

### Reporting summary

Further information on experimental design is available in the Nature Research Reporting Summary linked to this article.

## Supplementary information

Supplementary Information

Description of Additional Supplementary Files

Supplementary Movie 1

Supplementary Movie 2

Supplementary Movie 3

Supplementary Movie 4

Supplementary Movie 5

Supplementary Movie 6

Supplementary Movie 7

Supplementary Data 1

Supplementary Data 2

Reporting Summary

## Data Availability

Data on proteomic analysis described here are available on PRIDE ‘PXD011730’. All relevant data that support the findings of this study are available from the corresponding author upon reasonable request.
