# Author Correction: Loss-of-function mutations in *QRICH2* cause male infertility with multiple morphological abnormalities of the sperm flagella

**DOI:** 10.1038/s41467-019-10313-x

**Published:** 2019-05-20

**Authors:** Ying Shen, Feng Zhang, Fuping Li, Xiaohui Jiang, Yihong Yang, Xiaoliang Li, Weiyu Li, Xiang Wang, Juan Cheng, Mohan Liu, Xueguang Zhang, Guiping Yuan, Xue Pei, Kailai Cai, Fengyun Hu, Jianfeng Sun, Lanzhen Yan, Li Tang, Chuan Jiang, Wenling Tu, Jinyan Xu, Haojuan Wu, Weiqi Kong, Shuying Li, Ke Wang, Kai Sheng, Xudong Zhao, Huanxun Yue, Xiaoyu Yang, Wenming Xu

**Affiliations:** 1 Department of Obstetrics/Gynecology, Joint Laboratory of Reproductive Medicine (SCU-CUHK), Key Laboratory of Obstetric, Gynecologic and Pediatric Diseases and Birth Defects of Ministry of Education, West China Second University Hospital, Sichuan University, 610041 Chengdu, China; 2 Obstetrics and Gynecology Hospital, Institute of Metabolism and Integrative Biology, School of Life Sciences, Fudan University, 200011 Shanghai, China; 3 State Key Laboratory of Reproductive Medicine, Center for Global Health, School of Public Health, Nanjing Medical University, 211116 Nanjing, China; 4 Shanghai Key Laboratory of Female Reproductive Endocrine Related Diseases, 200011 Shanghai, China; 5 Human Sperm Bank, West China Second University Hospital, Sichuan University, 610041 Chengdu, China; 6 Key Laboratory of Birth Defects and Related Disease of Women and Children (Sichuan University), Ministry of Education, 610041 Chengdu, China; 7 Center of Reproductive Medicine, West China Second University Hospital, Sichuan University, 610041 Chengdu, China; 8 Southwest Medical University, 646000 Luzhou, China; 9 Department of Medical Genetics, State Key Laboratory of Biotherapy, West China Hospital, Sichuan University and Collaborative Innovation Center, 610041 Chengdu, China; 10 Analytical & Testing Center, Sichuan University, 610041 Chengdu, China; 11 Chengdu Research Base of Giant Panda Breeding, 610041 Chengdu, China; 12 Auckland University, 1142 Auckland, New Zealand; 13 Teaching Hospital of Chengdu University of TCM, 610041 Chengdu, China; 14 Key Laboratory of Animal Models and Human Disease Mechanisms of Chinese Academy of Sciences and Yunnan Province, Kunming Institute of Zoology, Chinese Academy of Sciences, 650000 Kunming, China; 15 Center for Excellence in Animal Evolution and Genetics, Chinese Academy of Sciences, 650223 Kunming, China; 16 State Key Laboratory of Reproductive Medicine, Clinical Center of Reproductive Medicine, The First Hospital, Nanjing Medical University, 210029 Nanjing, China

Correction to: *Nature Communications* 10.1038/s41467-018-08182-x, published online 25 January 2019.

In the original version of this article, the Acknowledgements section omitted the National Key Research and Development Program of China (SQ2018YFC1003603).

This error has now been corrected in both the PDF and HTML versions of the article.

