# Supplementary material for: Loss-of-function mutations in QRICH2 cause male infertility with multiple morphological abnormalities of the sperm flagella
Source: Nat Commun. 2019 Jan 25;10:433. doi: 10.1038/s41467-018-08182-x (PMC6347614; doi:10.1038/s41467-018-08182-x)
Supplement: Supplementary file 1 — Supplementary Information [file 41467_2018_8182_MOESM1_ESM.pdf]

Loss-of-function mutations in *QRICH2* cause male infertility with multiple morphological abnormalities of the sperm flagella

Ying Shen et al.

Supplementary Information

Supplementary Figure 1

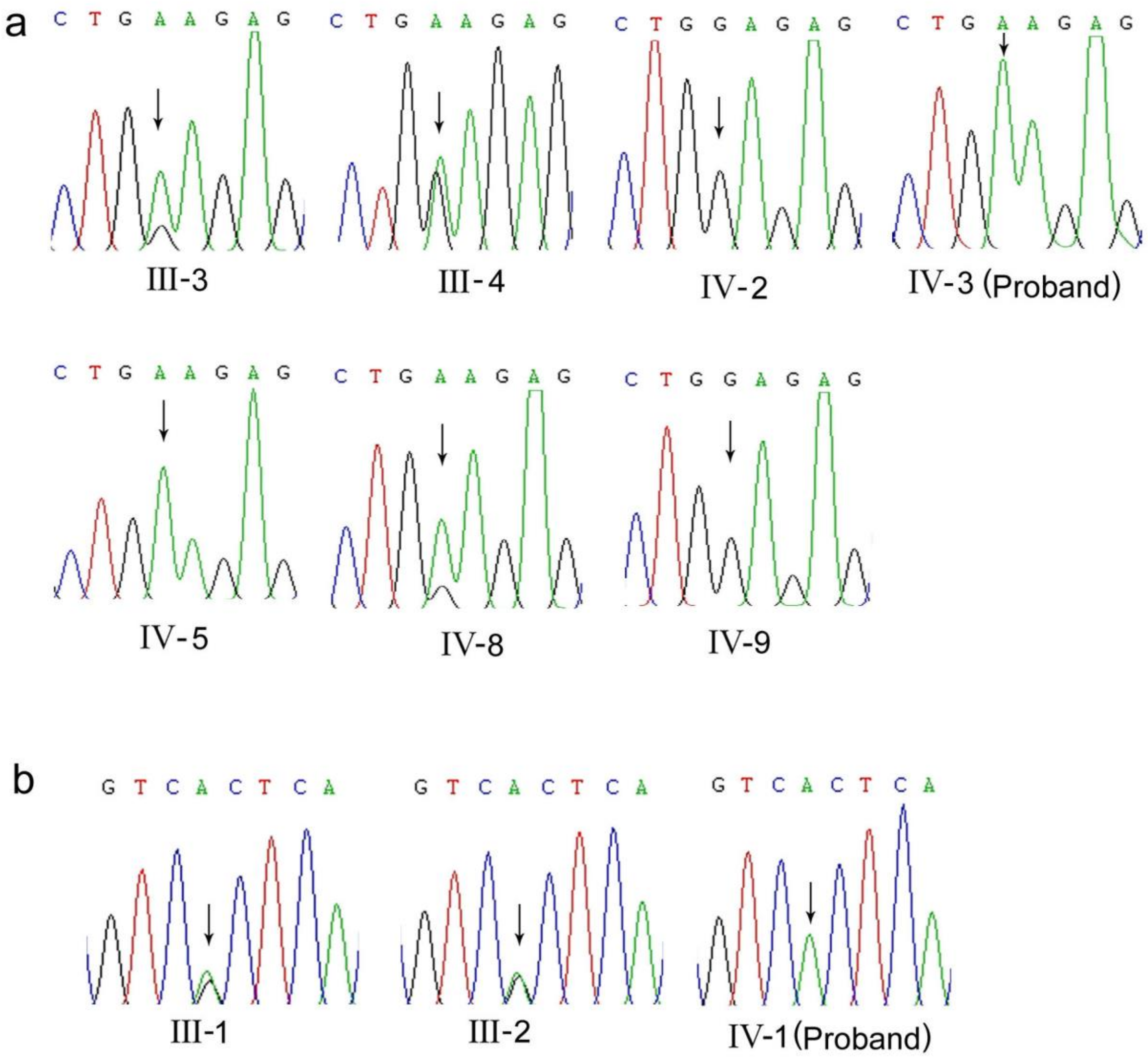

Supplementary Fig 1. Sequence chromatograms of two consanguineous families. **a** The base status of the mutation (c.192G>A) with proband in family A. **b** The base status of the mutation (c.3037C>T) with proband in family B.

Supplementary Figure 2

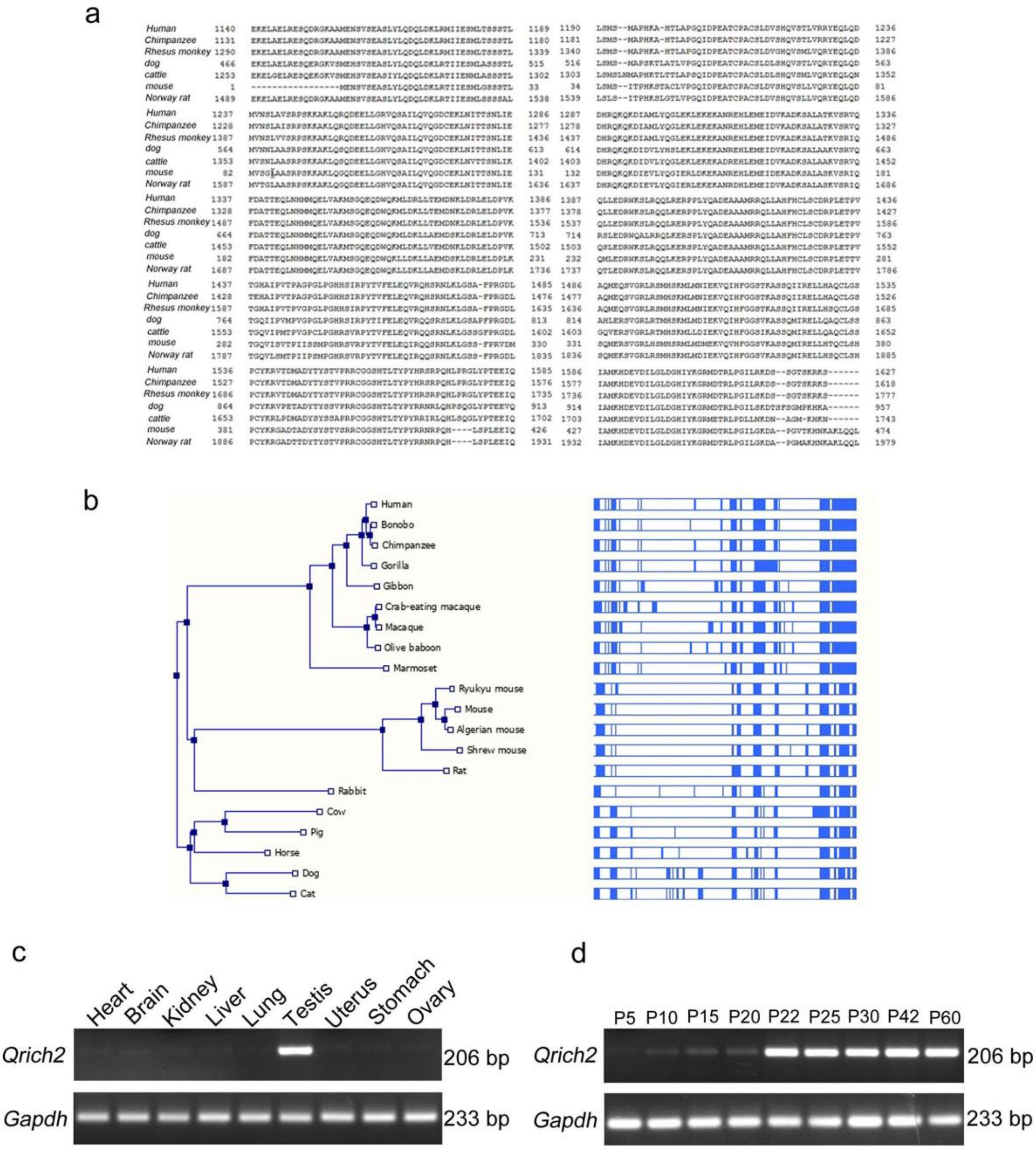

Supplementary Fig 2. Qrich2 is a testis-enriched gene during evolution. **a** The high homology of the QRICH2 protein sequence in several species. **b** The multi-alignment analyses of QRICH2 orthologs in 20 species. **c** Qrich2 expression in multiple tissues of mice. The real time PCR results suggested that Qrich2 is exclusively detected in the testes. **d** Qrich2 expression in the testes of various postnatal days. Stable and high expression of Qrich2 was detected in the P22 and afterward testes by real time PCR.

Supplementary Figure 3

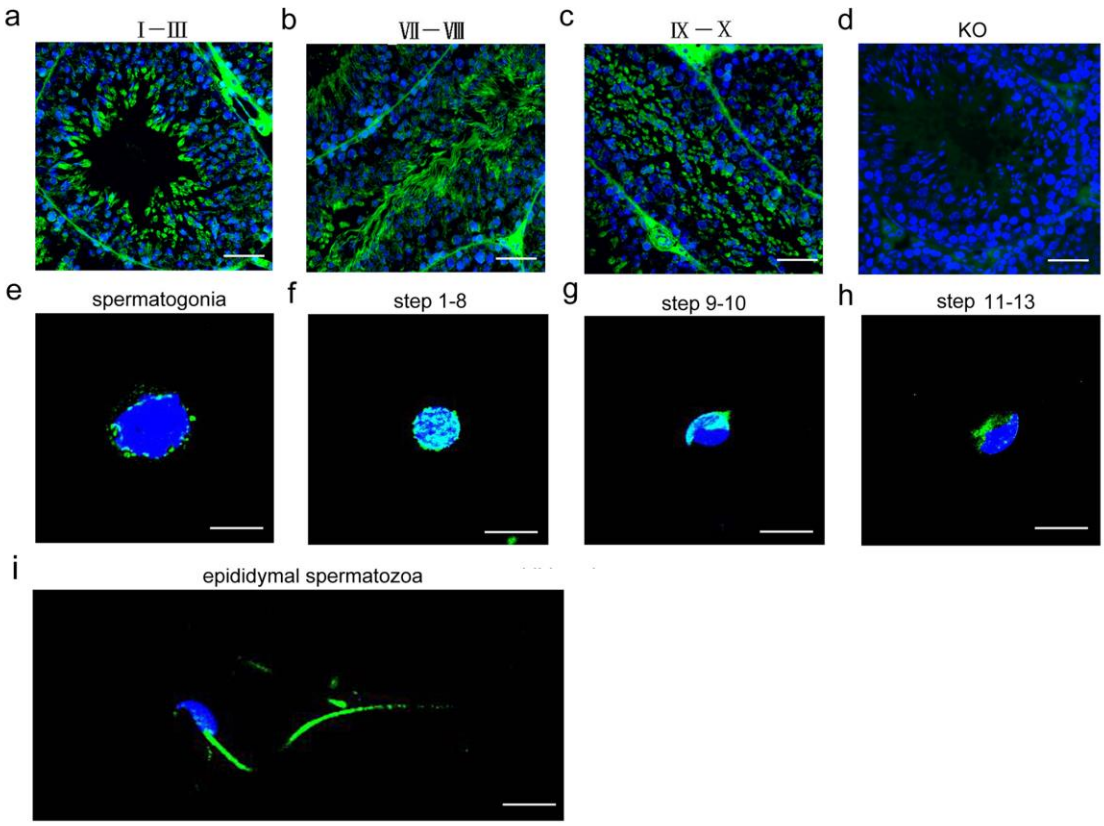

Supplementary Fig 3. The expression of Qrich2 in mice testes tissues. **a-d** the immunofluoresence results of mice testes section from WT mice showed that Qrich2 is primarily localized in the round and elongated spermatids during different spermiogenesis stages (**a**-**c**), and Qrich2 is not detectable in the testes tissues of KO mice (**d**) (green, Qrich2; blue, DAPI; scale bars, 20 µm). **e-i** Qrich2 is expressed in the nuclear membrane of the spermatogonia (**e**), in the nucleus of the round spermatids (step 1-8) (**f**), in the nucleus and cytoplasm of the early elongating spermatids (step 9-10) (**g**), in the cytoplasm of late elongating spermatids (step 11-13) (**h**) and in the flagella of epididymal spermatozoa (**i**) (green, Qrich2; blue, DAPI; scale bars, 5 µm).

Supplementary Figure 4

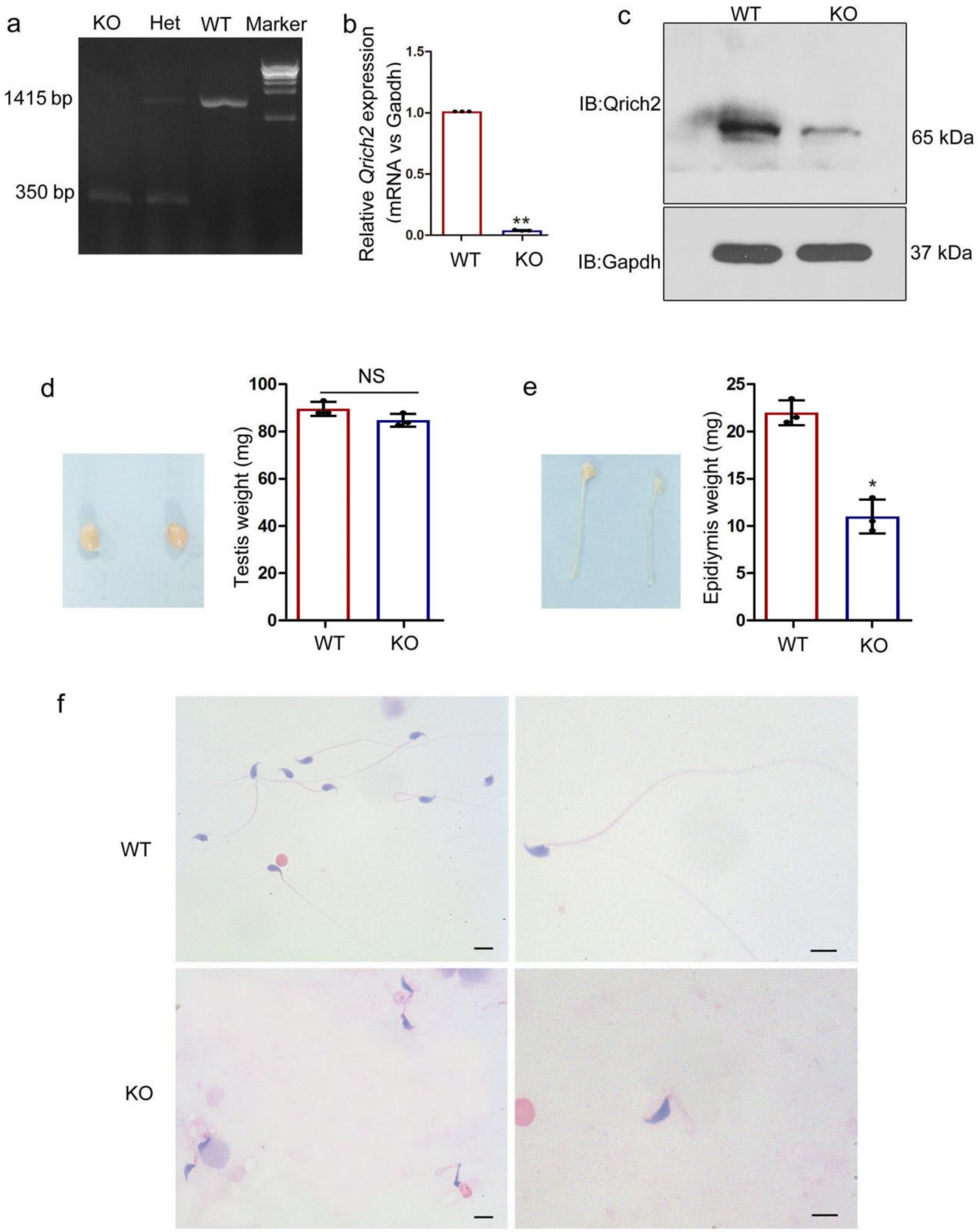

Supplementary Fig 4. Defects of fertility in male mice caused by inaction of Qrich2.

a The result of PCR-based genotyping using ear DNA. **b** Extremely low mRNA levels

of *Qrich2* in the testes of the KO mice compared with that in the WT mice (Student's *t*-test; n = 3 independent experiments; $^{*}p < 0.01$; error bars, s.e.m). **c** Qrich2 protein levels in the testes of the WT and KO mice. **d** The similar testis weights for the WT and KO mice (Student's *t*-test; n = 3 biologically independent WT mice or KO mice; NS, not significant; error bars, s.e.m). **e** The epididymis weight of the KO mice is less than that of the WT mice (Student's *t*-test; n = 3 biologically independent WT mice or KO mice; $^{*}p < 0.05$; error bars, s.e.m). **f** The flagellar abnormalities in the KO mice compared with the WT mice by light microscopy (scale bars, 5 μm).

Supplementary Figure 5

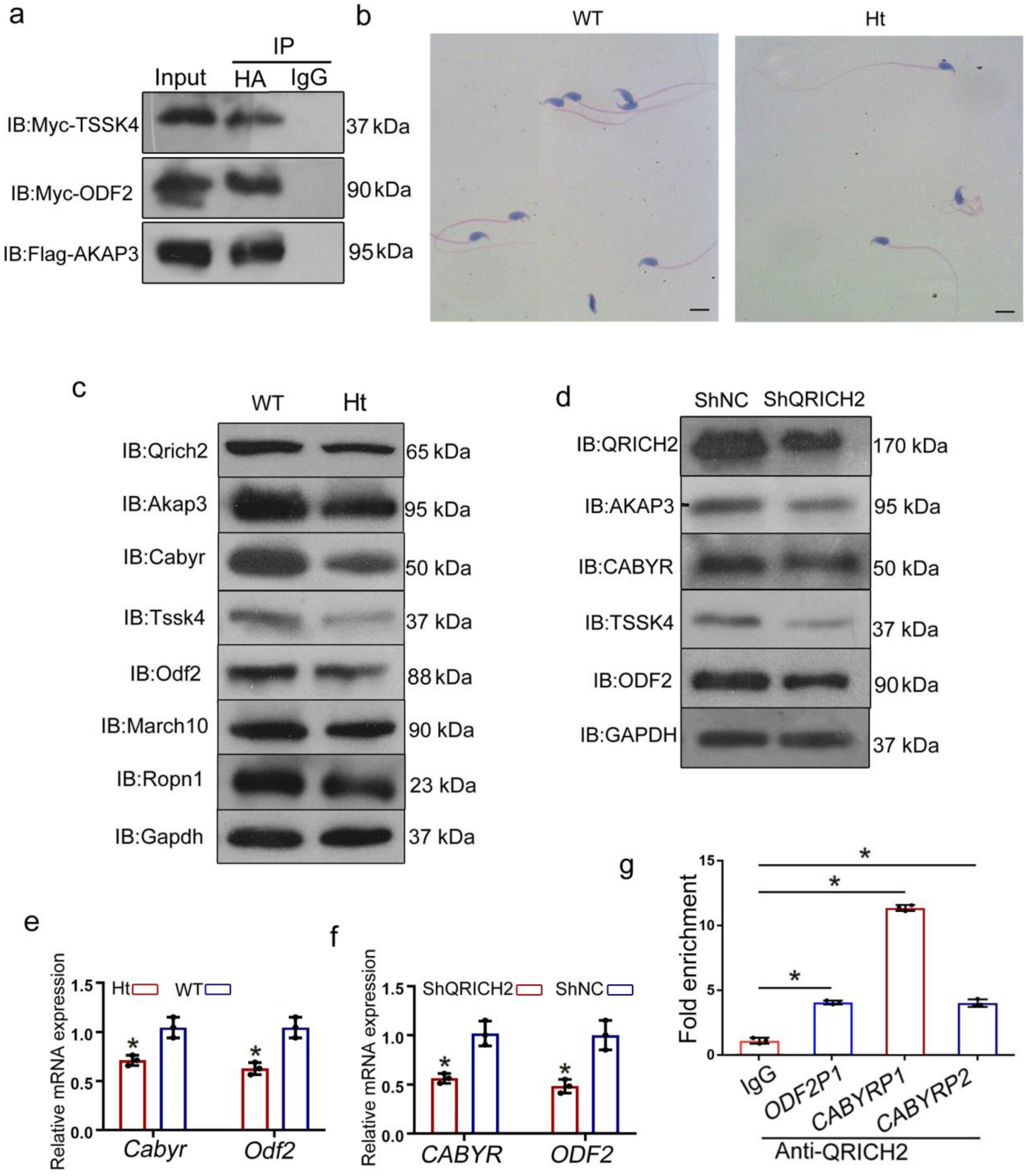

Supplementary Fig 5. QRICH2 regulated the expression of AKAP3, CABYR, TSSK4, ODF2, MARCH10 and ROPN1. **a** The HA-QRICH2 plasmid was transfected with Flag-AKAP3, Myc-TSSK4, Myc-ODF2 plasmids respectively in HEK 293 cells. The Co-IP results showed that QRICH2 could bind to AKAP3, ODF2 and TSSK4. **b**

The morphology of sperm flagella in Ht mice are as normal as the WT mice (scale bars, 5 μm). **c** The reduced protein levels of Akap3, Cabyr, Tssk4, Odf2, March10 and Ropn1 were observed in Ht mice testes compared with WT mice testes. **d** The expression of AKAP3, CABYR, TSSK4 and ODF2 was decreased in NT2 cells with QRICH2 knocked down. **e** The real time PCR results showed the reduced mRNA levels of *Odf2* and *Cabyr* in Ht mice testes compared with WT mice testes (Student's t-test; n = 3 independent experiments; $^{*}p < 0.01$; error bars, s.e.m). **f** Knocking down QRICH2 in NT2 cells down-regulated the expression of *ODF2* and *CABYR* in mRNA levels (Student's t-test; n = 3 independent experiments; $^{*}p < 0.01$; error bars, s.e.m). **g** ChIP-qPCR results showed the binding of QRICH2 to *ODF2* and *CABYR* promoter fragments (Student's t-test; n = 3 independent experiments; $^{*}p < 0.01$; error bars, s.e.m).

Supplementary Table

Supplementary Table 1. Analysis of *QRICH2* mutations in controls

	AA	128		AA	71		AA	23		CC	25		AA	28
rs443970	AG	52	rs7225131	AT	94	rs6501883	AG	106	rs6501882	CT	104	rs6501881	AG	101
	GG	20		TT	35		GG	71		TT	71		GG	71
	AA	26		CC	3		AA	2		AA	64		CC	58
rs6501880	AG	103	rs8068457	CG	11	rs73996306	AG	12	rs2279054	AG	91	rs2279053	CT	98
	GG	71		GG	186		GG	186		GG	45		TT	44
	CC	65		CC	30		CC	25		AA	29		AA	189
rs2279052	CG	87	rs4789273	CT	93	rs4789274	CT	97	rs17509527	AG	61	rs67948583	AG	9
	GG	48		TT	77		TT	78		GG	110		GG	2
	CC	65		AA	0		AA	1		AA	15		AA	1
rs164011	CT	130	rs73999020	AG	4	rs73350377	AG	3	rs164010	AG	96	rs73350386	AG	5
	TT	5		GG	196		GG	196		GG	89		GG	194
	AA	0		AA	160		AA	0		AA	0		AA	0
rs143270448	AG	2	rs343675	AG	31	c.1248G>A p.Q416Q	AG	11	c.1254T>A p.G418G	AT	12	c.1098G>A p.Q366Q	AG	5
	GG	198		GG	9		GG	189		TT	188		GG	195

Supplementary Table 2. Semen analysis in wild-type (WT) and heterozygous (Ht) male mice by CASA

	Sample	
	WT	Ht
Semen Parameters		
Sperm concentration (10^6/ml)†*	25.77±2.54	19.21±2.06
Motility (%)*	47.23±4.52	20.26±4.6
Progressive motility (%)*	37.26±3.03	14.53±2.83
Sperm locomotion parameters		
Curvilinear velocity (VCL) (μm/s)*	100.24±4.59	34.92±2.97
Straight-line velocity (VSL) (μm/s)*	25.53±0.95	9.30±0.68
Average path velocity (VAP) (μm/s)*	40.32±1.20	13.88±1.16
Amplitude of lateral head displacement (ALH) (μm)*	1.31±0.047	0.49±0.067
Linearity (LIN)	0.25±0.02	0.29±0.09
Wobble (WOB, =VAP/VCL)	0.40±0.02	0.43±0.06
Straightness (STR, =VSL/VAP)	0.63±0.01	0.68±0.08
Beat-cross frequency (BCF) (Hz)*	7.46±0.55	2.70±0.36

*A significant difference $P < 0.05$ (n=4), Student's t-test

†Epididymides and vas deferens

Supplementary Table 3. The information regarding the PCR primers used in the present study

Target	Forward primer（5’- 3’）	Reverse primer（5’-3’）	Product （bp）
Q (c.192G>A)	TTCACTGGTCACTTGAAGGG	CATTGATGGTGGGGGTCTAC	533
Q (c.3037C>T)	CTGGGGCTTAGTTCTCTCCT	CCCAATACCACTGAGTACA	729
Q exon 1	CACGTGCAGCACTTCTCC	TAGGCCTTTTGGTGGCGT	383
Q exon2-exon3	GCGTGTGTAGGGGCCACT	CAGAATCCTGTGCAGCTGTA	661
Q exon 4-1	CTCAAGTAATCCACCCGCCT	ATTCCCGATCATCCATTTCA	784
Q exon 4-2	ATGGTGTGGTACCCCTCA	GTACCAAGACACCCTGACC	761
Q exon 4-3	GGTCAGCATGATTTGGTC	GTGGTATTGGGCTGTGCT	1257
Q exon 4-4	TTGGCATCACCTGGTATAGA	GACTCTGAAGGCTGTGGGAC	782
Q exon 5	AGGGGCTGGAAATGGAGATT	CTGAGGCATGAGAATCGCTT	433
Q exon 6	AGGGGGTTGGGTTAGTAGAG	GACACAATCCAGGTCAAAGA	343
Q exon 7	GAGTGTGGAGTCTGGCAGTC	CTTGGTCCCTGGAGCTAAAG	189
Q exon 8	TGAGGATGCAGCAACTGCC	CAGGGTGGCCCTCCTCTGT	445
Q exon9-exon10	AGCAGCCTCCGAGAGCCT	TCCCCTCCACTCAGTCTCTC	422
Q exon11-exon12	GGCCATCTCCCAGATGCG	CTAAAATTCTGTCCCTCCAACC	615
Q exon13-exon14	TCTTCCGGGCAGAGCACTC	CCTTCTGTCACGTTCCGCT	548
Q exon 15	GGAACTGAAGGATTCGGCTTA	GCCCACACTTTCTCTCACA	425
Q exon 16	CCCCCGAAGAGAAGCTGTG	TTGCCTCAAGGCAGAGGGC	374
Q exon 17	AGGAGGGTTCTGAGACCACAG	GTCTAGTGGCCCATAAAAGTGC	287

Q exon 18	TTCCCTCCTGTCCCCCATC	CTTTTCCACACCCATCCCC	288
mice genotype	GGCAGAGGCAGCAGGAGATAG	CACTTGGTCCATGGCTGTTGATT	1415/350
CABYR promoter	GAAATCAAAGCAGCGATGGGC	CCCACCCCCGACTCTGAGGAC	2051
ODF2 promoter	TTTTTTTAAGAGATTGGGCCT	CCTCCGCGCCCGGTCTACTAC	1967
CABYR P1	CAGAAATCATGACAGCCTCATC	AAAGCAATGTTAAGGCCTCAGT	287
CABYR P2	TGAATGGCACCAGATAATGC	CAGGGCAGATATCTGACCAA	160
ODF2 P1	CTGCATAGTCTGGTCCTGCTGA	GCCATGAAAGGTGAGTGGAAGT	200

Supplementary Table 4. The information regarding the real time PCR primers used in the present study

Target	Forward primer（5’-3’）	Reverse primer（5’-3’）	Product（bp）
Qrich2	CTCTGTCTCTGAGGCGTCTC	GCAGACTGACTTGGTGGCTC	206
Akap3	GTTACAAAGTCAAAGTGGCGT	ACTGGGTAAGCCACTTCCTCC	207
Odf2	GCTGAAGCTTTATCTACTCTGG	CCTGGCTCTTGTAGTTGTCTAT	346
Tssk4	GCGGGAGAATGTGAAGATA	AGGTTCTTGCACTCCTGGG	336
Ropn1	AGTGAGAGAGAGGTCTGAACA	TGAGAGTTTTGGCAATGGTAAC	263
Cabyr	GATGAAAGTAGAGAAATGGGC	ACTCGGCAGAGACAGGCG	305
March10	AAGAGTGCCTGAAAAAGTGGC	CGAACCTCTGCTCATATAGGTG	222
Gapdh	GGTGAAGGTCGGTGTGAACG	CTCGCTCCTGGAAGATGGTG	233

Supplementary Methods

1. Protein Extraction

The mouse testes were grinded by liquid nitrogen into cell powder and then transferred to a 5-mL centrifuge tube. After that, four volumes of lysis buffer (8 M urea, 1% Protease Inhibitor Cocktail) was added to the cell powder, followed by sonication three times on ice using a high intensity ultrasonic processor (Scientz). The remaining debris was removed by centrifugation at 12,000g at 4 °C for 10 min. Finally, the supernatant was collected and the protein concentration was determined with BCA kit according to the manufacturer's instructions.

2. Trypsin Digestion

For digestion, the protein solution was reduced with 5 mM dithiothreitol for 30 min at 56 °C and alkylated with 11 mM iodoacetamide for 15 min at room temperature in darkness. The protein sample was then diluted by adding 100 mM TEAB to urea concentration less than 2M. Finally, trypsin was added at 1:50 trypsin-to-protein mass ratio for the first digestion overnight and 1:100 trypsin-to-protein mass ratio for a second 4h digestion.

3. TMT Labeling

After trypsin digestion, peptide was desalted by Strata X C18 SPE column (Phenomenex) and vacuum-dried. Peptide was reconstituted in 0.5 M TEAB and processed according to the manufacturer's protocol for TMT kit. Briefly, one unit of TMT reagent were thawed and reconstituted in acetonitrile. The peptide mixtures were then incubated for 2h at room temperature and pooled, desalted and dried by

vacuum centrifugation.

4. HPLC Fractionation

The tryptic peptides were fractionated into fractions by high pH reverse-phase HPLC using Agilent 300Extend C18 column (5 μm particles, 4.6 mm ID, 250 mm length). Briefly, peptides were first separated with a gradient of 8% to 32% acetonitrile (pH 9.0) over 60 min into 60 fractions. Then, the peptides were combined into 18 fractions and dried by vacuum centrifuging.

5. LC-MS/MS Analysis

The tryptic peptides were dissolved in 0.1% formic acid (solvent A), directly loaded onto a home-made reversed-phase analytical column (15-cm length, 75 μm i.d.). The gradient was comprised of an increase from 6% to 23% solvent B (0.1% formic acid in 98% acetonitrile) over 26 min, 23% to 35% in 8 min and climbing to 80% in 3 min then holding at 80% for the last 3 min, all at a constant flow rate of 400 nL/min on an EASY-nLC 1000 UPLC system.

The peptides were subjected to NSI source followed by tandem mass spectrometry (MS/MS) in Q Exactive^{TM} Plus (Thermo) coupled online to the UPLC. The electrospray voltage applied was 2.0 kV. The m/z scan range was 350 to 1800 for full scan, and intact peptides were detected in the Orbitrap at a resolution of 70,000. Peptides were then selected for MS/MS using NCE setting as 28 and the fragments were detected in the Orbitrap at a resolution of 17,500. A data-dependent procedure that alternated between one MS scan followed by 20 MS/MS scans with 15.0s dynamic exclusion. Automatic gain control (AGC) was set at 5E4. Fixed first mass

was set as 100 m/z.

6. Data Analysis

The raw data were processed by GO Annotation (www. http://www.ebi.ac.uk/GOA/), Domain Annotation (InterProScan) and KEGG Pathway Annotation (KEGG online service tools KAAS mapper).

Full scans

Fig.5d

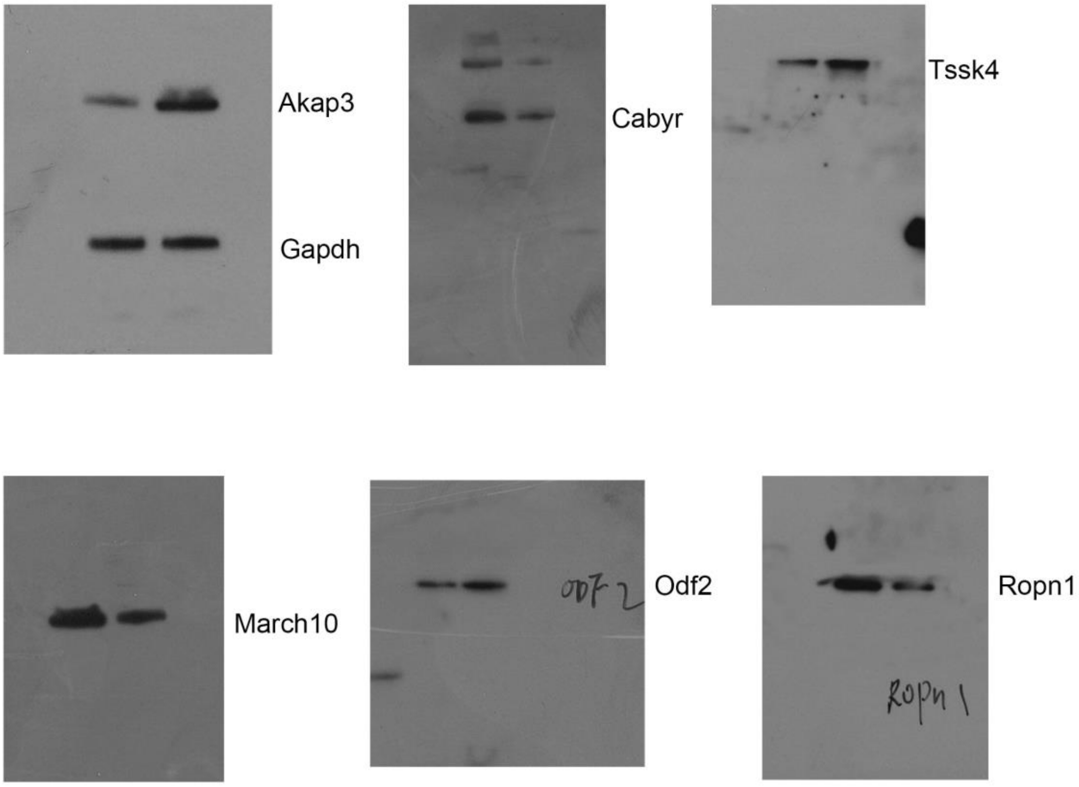

Fig.6b

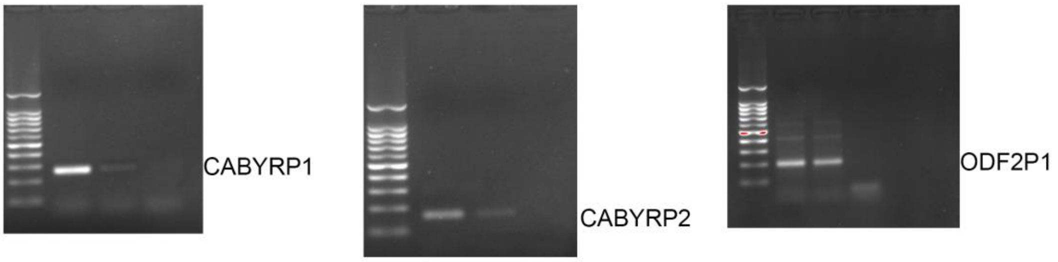

Fig.6e

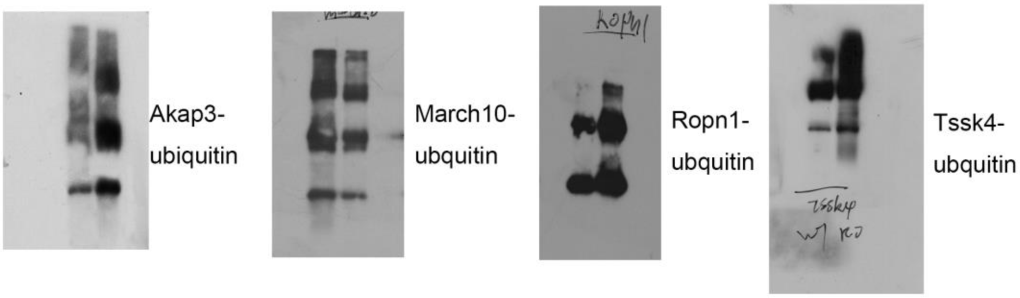

Fig.6f

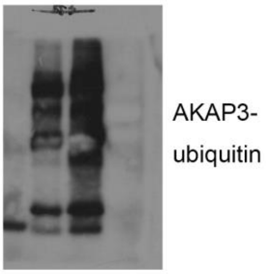

Fig.6g

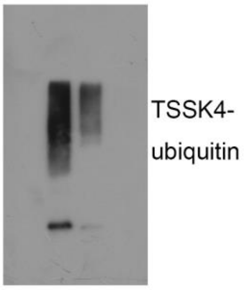

Fig.6h

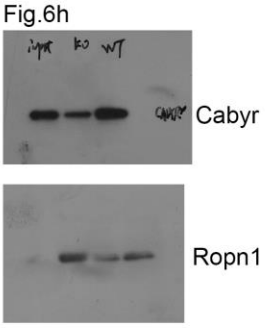

Fig.6i

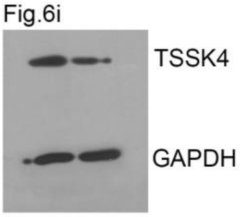

Fig.S2c

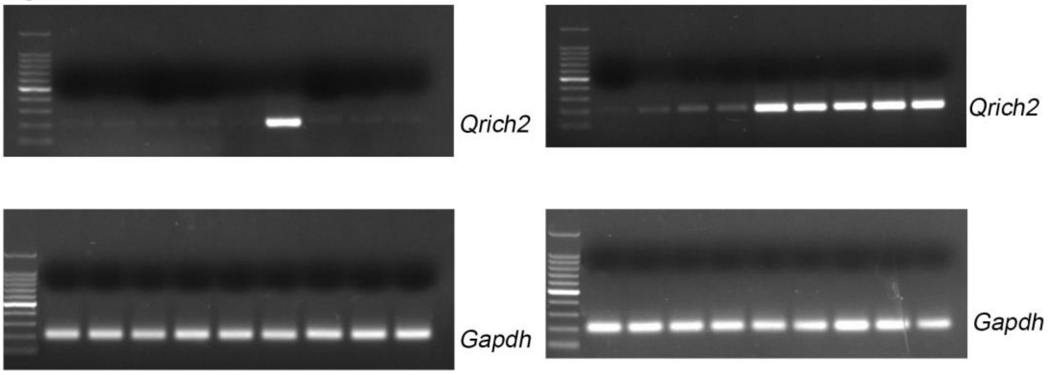

Fig.S6a

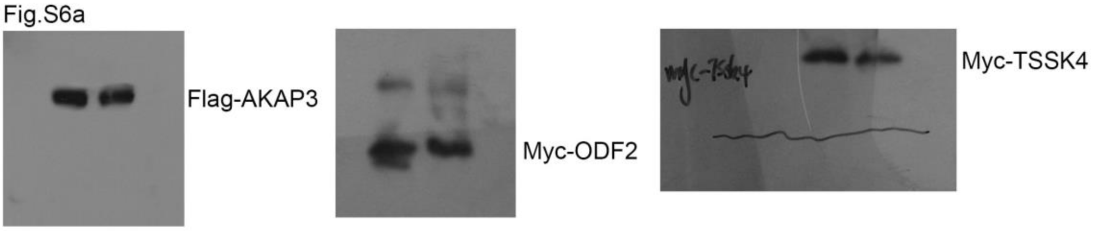

Fig.S6c

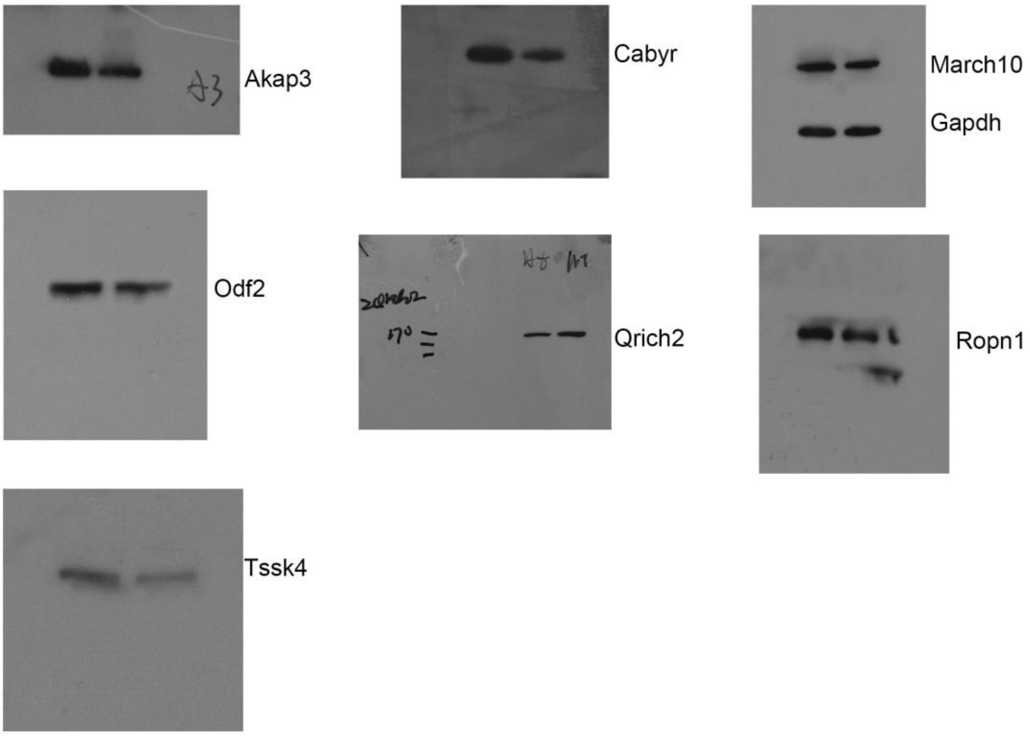

Fig.S6d

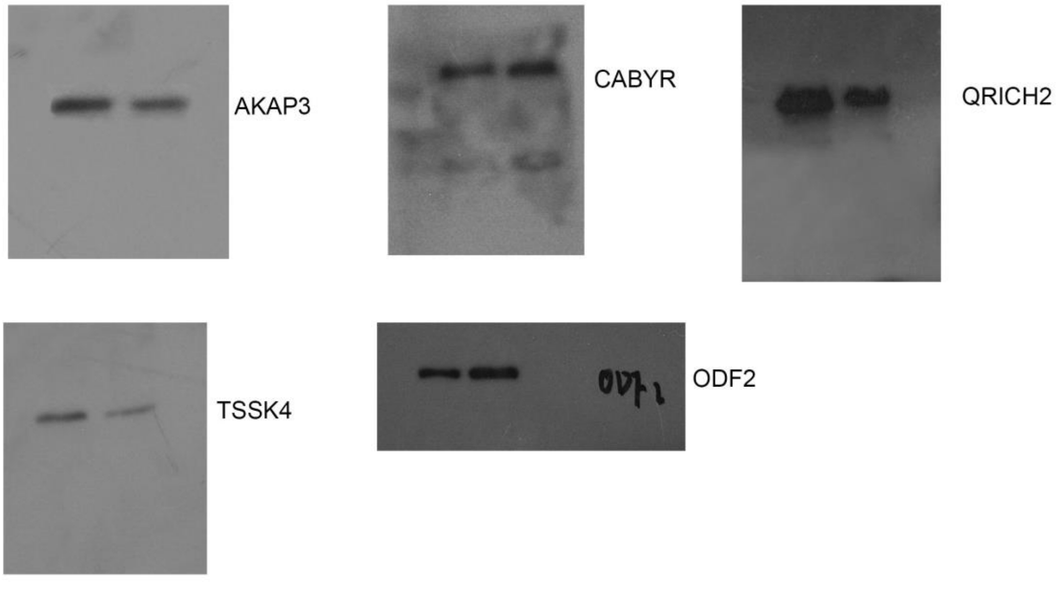

Figure exemplifying the gating strategy

WT

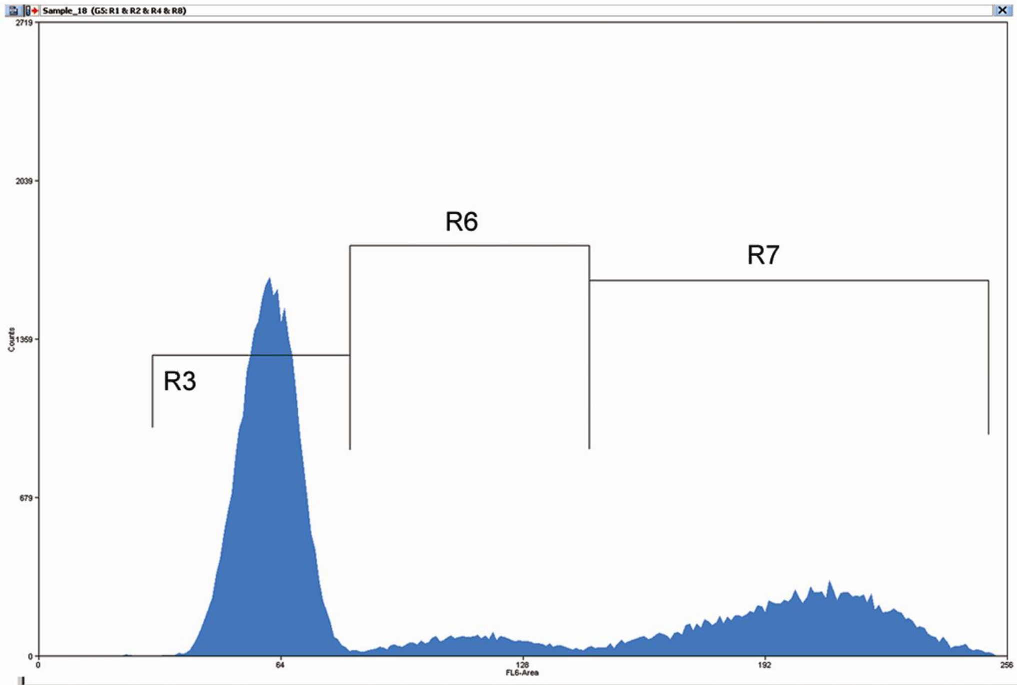

Region	Count	% Hist	% All	Bounds	Mode Count	Mode	Mean	Median	Std Dev.	CV	CV (hm)	Skew
Total	50000	100.00	45.16	(0.00,25...	1622	61.00	108.42	67.00	65.66	60.56	13.20	0.78
R6	3505	7.01	3.17	(82.00,1...	102	120.00	114.83	115.00	15.17	13.21	11.65	-0.07
R7	15661	31.32	14.14	(145.00,...	324	209.00	200.92	203.00	22.54	11.22	9.33	-0.31
R3	30853	61.71	27.87	(30.00,8...	1622	61.00	60.66	61.00	7.45	12.28	13.20	-0.09

KO

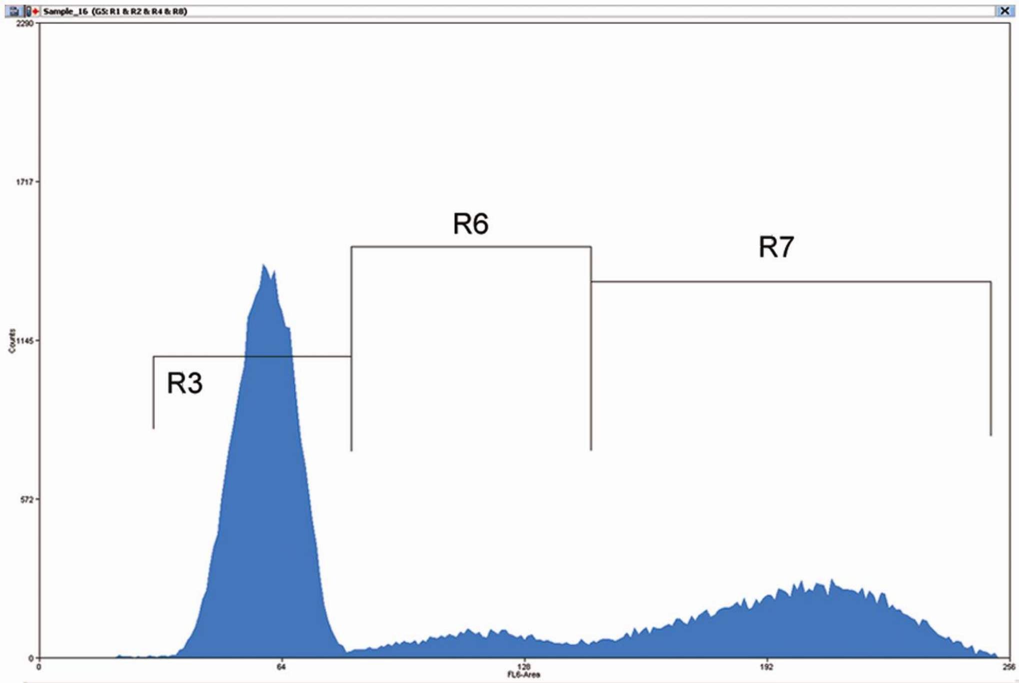

Region	Count	% Hist	% All	Bounds	Mode Count	Mode	Mean	Median	Std Dev.	CV	CV (hm)	Skew
Total	50000	100.00	19.56	(0.00,25...	1419	59.00	109.84	68.00	65.97	60.05	15.08	0.70
R6	4135	8.27	1.62	(82.00,1...	106	113.00	115.71	116.00	15.98	13.81	13.87	-0.11
R7	16419	32.84	6.42	(145.00,...	284	209.00	198.73	201.00	23.77	11.96	11.76	-0.21
R3	29445	58.89	11.52	(30.00,8...	1419	59.00	59.48	60.00	8.01	13.46	15.08	-0.13
