# Supplementary material for: Loss-of-function mutations in QRICH2 cause male infertility with multiple morphological abnormalities of the sperm flagella
Source: Nat Commun. 2019 Jan 25;10:433. doi: 10.1038/s41467-018-08182-x (PMC6347614; doi:10.1038/s41467-018-08182-x)
Supplement: Supplementary file 2 — Description of Additional Supplementary Files [file 41467_2018_8182_MOESM2_ESM.pdf]

Description of Additional Supplementary Files

File Name: Supplementary Movie 1
Description: 3D Representations of QRICH2 on the sperm flagella, Related to Fig 2a.

File Name: Supplementary Movie 2
Description: 3D Representations of α-Tubulin on the sperm flagella, Related to Fig 2b.

File Name: Supplementary Movie 3
Description: 3D Representations of the merge of QRICH2 and α-Tubulin on the sperm flagella, Related to Fig 2c.

File Name: Supplementary Movie 4
Description: The WT epididymal sperm were collected into the HTF medium, incubated at 37°C for 10 min and loaded onto a chamber slide for video recording under a phase-contrast microscope. Normal quantity and motility were observed (n=4 biologically independent WT mice).

File Name: Supplementary Movie 5
Description: Few sperm could be collected from the cauda epididymis and vas deferens of Qrich2 KO male mice. Furthermore, the reduced motility and abnormal flagellar morphology were observed (n=4 biologically independent KO mice).

File Name: Supplementary Movie 6
Description: The reduced motility of sperm from the cauda epididymis and vas deferens was observed in Ht mice compared with WT mice (Supplementary Movie 7) (n=4 biologically independent Ht mice).

File Name: Supplementary Movie 7
Description: The normal motility of sperm from the cauda epididymis and vas deferens was observed in WT mice (n=4 biologically independent WT mice).

File Name: Supplementary Data 1
Description: The homozygous variants found in the two patients by WES analysis.

File Name: Supplementary Data 2
Description: The sperm parameters of asthenozoospermia subjects.
