# Supplementary material for: Loss-of-function mutations in QRICH2 cause male infertility with multiple morphological abnormalities of the sperm flagella
Source: Nat Commun. 2019 Jan 25;10:433. doi: 10.1038/s41467-018-08182-x (PMC6347614; doi:10.1038/s41467-018-08182-x)
Supplement: Supplementary file 12 — Reporting Summary [file 41467_2018_8182_MOESM12_ESM.pdf]

nature research

Corresponding author(s): Wenming Xu NCOMMS-18-10636B

Reporting Summary

Nature Research wishes to improve the reproducibility of the work that we publish. This form provides structure for consistency and transparency in reporting. For further information on Nature Research policies, see Authors & Referees and the Editorial Policy Checklist.

Statistical parameters

When statistical analyses are reported, confirm that the following items are present in the relevant location (e.g. figure legend, table legend, main text, or Methods section).

n/a	Confirmed	
☐	☒	The exact sample size (*n*) for each experimental group/condition, given as a discrete number and unit of measurement
☐	☒	An indication of whether measurements were taken from distinct samples or whether the same sample was measured repeatedly
☐	☒	The statistical test(s) used AND whether they are one- or two-sided<br>*Only common tests should be described solely by name; describe more complex techniques in the Methods section.*
☒	☐	A description of all covariates tested
☒	☐	A description of any assumptions or corrections, such as tests of normality and adjustment for multiple comparisons
☐	☒	A full description of the statistics including central tendency (e.g. means) or other basic estimates (e.g. regression coefficient) AND variation (e.g. standard deviation) or associated estimates of uncertainty (e.g. confidence intervals)
☐	☒	For null hypothesis testing, the test statistic (e.g. *F*, *t*, *r*) with confidence intervals, effect sizes, degrees of freedom and *P* value noted<br>*Give P values as exact values whenever suitable.*
☒	☐	For Bayesian analysis, information on the choice of priors and Markov chain Monte Carlo settings
☒	☐	For hierarchical and complex designs, identification of the appropriate level for tests and full reporting of outcomes
☒	☐	Estimates of effect sizes (e.g. Cohen's *d*, Pearson's *r*), indicating how they were calculated
☐	☒	Clearly defined error bars<br>*State explicitly what error bars represent (e.g. SD, SE, CI)*

Our web collection on statistics for biologists may be useful.

Software and code

Policy information about availability of computer code

Data collection	EXCEL 2016,SPSS 17.0
Data analysis	Graphpad Prism 6,SPSS17.0 , PyMol,SWISS-MODEL

For manuscripts utilizing custom algorithms or software that are central to the research but not yet described in published literature, software must be made available to editors/reviewers upon request. We strongly encourage code deposition in a community repository (e.g. GitHub). See the Nature Research guidelines for submitting code & software for further information.

Data

Policy information about availability of data

All manuscripts must include a data availability statement. This statement should provide the following information, where applicable:

- Accession codes, unique identifiers, or web links for publicly available datasets
- A list of figures that have associated raw data
- A description of any restrictions on data availability

Data on proteomic analysis described here are available on PRIDE 'PXD011730 [(https://www.ebi.ac.uk/pride/archive/projects/PXD011730)]'
. All relevant data that support the findings of this study are available from the corresponding author upon reasonable request.

Field-specific reporting

Please select the best fit for your research. If you are not sure, read the appropriate sections before making your selection.

☒ Life sciences ☐ Behavioural & social sciences ☐ Ecological, evolutionary & environmental sciences

For a reference copy of the document with all sections, see nature.com/authors/policies/ReportingSummary-flat.pdf

Life sciences study design

All studies must disclose on these points even when the disclosure is negative.

Sample size	The minimal sample size was decided based on our preliminary data we got from the current project to ensure a a statistical power at the level (1-beta) of 80% and a significant level (alpha) of 5% fot t-test.
Data exclusions	No specific data were exluded for the current data set.
Replication	For each experiment with in vivo mice model, at least three for each genotype of mice and the number of mice in each group were pooled from multiple experiments.
Randomization	All the in vitro culture works were randomized, and the no specific method of randomization was used.
Blinding	The genotypes of the mice were genotyped by the blinded to the investigators and the parameters were collected by the PCR

Reporting for specific materials, systems and methods

Materials & experimental systems

n/a	Involved in the study	
☒	☐	Unique biological materials
☐	☒	Antibodies
☐	☒	Eukaryotic cell lines
☒	☐	Palaeontology
☐	☒	Animals and other organisms
☐	☒	Human research participants

Methods

n/a	Involved in the study	
☒	☐	ChIP-seq
☐	☒	Flow cytometry
☒	☐	MRI-based neuroimaging

Antibodies

Antibodies used

anti-QRICH2 (sc-514279, Santa Cruz Biotechnology), anti-QRICH2 (HPA052219, Sigma-Aldrich) for immunofluorescence staining of mice sample, anti-QRICH2 (HPA021935, Sigma-Aldrich) for immunofluorescence staining of human sample, anti-AKAP3 (provided by Prof. Huayu Qi) for immunofluorescence staining, anti-AKAP3 (13907-1-AP, Proteintech) for Co-IP, anti-ODF2 (12058-1-AP, Proteintech), anti-CABYR (12351-1-AP, Proteintech), anti-ROPN1 (sc-130455, Santa Cruz Biotechnology), MARCH10 (bs-10732 R, Bioss), anti-TSSK4 (507861, ZSGB-BIO), anti-ubiquitin (ab7780, Abcam), anti-Flag Tag (66008-2-Ig, Proteintech), anti-Myc Tag (sc-764,Santa Cruz Biotechnolog), anti-α-Tubulin (T7451, Sigma-Aldrich) and anti-GAPDH (ab8245, Abcam). Anti-AKAP3 mouse monoclonal antibody for western blotting was kindly provided by Prof. Huayu Qi. Anti-TSSK4 rabbit polyclonal antibody for Co-IP was kindly provided by Prof. Long Yu.

Validation

The antibodies used in western blotting, Co-IP and immunofluorescence staining are as follows:
1. anti-QRICH2 (sc-514279, Santa Cruz Biotechnology)
(1) Species specificity: human
(2) Applications: WB, IF, IP
(3) validation: WB analysis in Jurkat and Hs 181 Tes whole cell lysates.
2. anti-QRICH2 (1:50, HPA052219, Sigma-Aldrich)
(1) Species specificity: human
(2) Applications: IHC, IF
(3) validation: orthogonal RNAseq
3. anti-QRICH2 (1:50, HPA021935, Sigma-Aldrich)
(1) Species specificity: human
(2) Applications: IHC
(3) validation: orthogonal RNAseq
4. anti-AKAP3 (1:100, 13907-1-AP, Proteintech)

(1) Species specificity: human, mouse, rat
(2) Applications: WB, IP, IHC, IF, ELISA
(3) validation: WB analysis for mouse testis tissue, IHC analysis for human testis tissue
(4)References：Young SA, et al. CABYR is essential for fibrous sheath integrity and progressive motility in mouse spermatozoa. J Cell Sci. 2016;129(23):4379-4387.
5. anti-ODF2 (1:500, 12058-1-AP, Proteintech)
(1) Species specificity: human, mouse, rat
(2) Applications: WB, IP, IHC, IF, ELISA
(3) validation: WB and IF analysis for mouse testis tissue
(4) References：Chung JJ, et al. Structurally distinct Ca (2+) signaling domains of sperm flagella orchestrate tyrosine phosphorylation and motility. Cell. 2014;157(4):808-22.)
6. anti-CABYR (1:500, 12351-1-AP, Proteintech)
(1) Species specificity: human, mouse
(2) Applications: WB, IHC, IF, ELISA
(3) validation: WB analysis for human and mouse testis tissue, IHC analysis for human testis tissue.
(4) References: Young SA, et al. CABYR is essential for fibrous sheath integrity and progressive motility in mouse spermatozoa. J Cell Sci. 2016 ;129(23):4379-4387.
7. anti-ROPN1 (1:200, sc-130455, Santa Cruz Biotechnology)
(1) Species specificity: human
(2) Applications: IHC(P), IF
8. MARCH10 (1:500, bs-10732 R ,Bioss)
(1) Species specificity: human, mouse, rat, dog, cow, horse, sheep
(2)Applications: WB, ELISA, IHC-P, IHC, ICC, IF
9. anti-TSSK4 (1:400, A7861, ABclonal)
(1) Species specificity: human, mouse
(2) Applications: WB
(3) validation: WB analysis for mouse testis tissue
10. anti-ubiquitin (1:1000, ab7780, Abcam)
(1) Species specificity: Mouse, Rat, Human, African green monkey
(2) Applications: ICC/IF, IHC-FoFr, IHC-P, WB, IP
(3) validation: WB analysis for human lung and pancreatic cancer cell line
(4) References: Li M et al. SUMO2 conjugation of PCNA facilitates chromatin remodeling to resolve transcription-replication conflicts. Nat. Commun. 2018; 9:2706.

Eukaryotic cell lines

Policy information about cell lines

Cell line source(s)	NT2 cells were obtained from the American Type Culture Collection
Authentication	Cell line authentication were performed by ATCC using COI assay.
Mycoplasma contamination	The cell lines have been tested negative for mycoplasma contamination
Commonly misidentified lines (See ICLAC register)	No cell lines used in this study were found in th database of commonly misidentified cell lines that is maintained by ICLAS and NCBI Biosample.

Animals and other organisms

Policy information about studies involving animals; ARRIVE guidelines recommended for reporting animal research

Laboratory animals	All animal experiments were performed in accordance with the recommendation of the Guide for the Care and Use of All animal experiments were performed in accordance with the recommendation of the Guide for the Care and Use of Laboratory Animals of the National Institutes of Health.
Wild animals	none.
Field-collected samples	none.

Human research participants

Policy information about studies involving human research participants

Population characteristics	200 unrelated Han Chinese males with normal fertility (fathering at least one offspring by natural fertilization) and sperm quality (sperm concentration≥15 million /mL; the percentage of progressively motile sperm≥32%) were recruited from volunteers served as the control group for the population study.
Recruitment	Two interfile males with MMAF from two consanguineous families were enrolled at the Human Sperm Bank of West China Second University Hospital of Sichuan University and the Center of Reproductive Medicine at the First Affiliated Hospital of Nanjing Medical University.

Flow Cytometry

Plots

Confirm that:

☒ The axis labels state the marker and fluorochrome used (e.g. CD4-FITC).

☒ The axis scales are clearly visible. Include numbers along axes only for bottom left plot of group (a 'group' is an analysis of identical markers).

☒ All plots are contour plots with outliers or pseudocolor plots.

☒ A numerical value for number of cells or percentage (with statistics) is provided.

Methodology

Sample preparation	The testes from 8-week-old male mice were removed and decapsulated. Firstly, the seminiferous tubules were incubated in 10 ml 1xPBS containing 90 mg/ml of collagenase (Invitrogen) with continuous agitation for 15 min at 32°C and then allowed to sediment for 5 min and the supernatant was removed. Next, the pellet was resuspended in 10 ml of PBS with 60 mg/ml of trypsin (Sigma-Aldrich) and 1 µg/ml of DNase (Promega), and incubated under the same conditions for 15 min. After gently being pipetted with a Pasteur pipette, the cell suspension was centrifuged at 400 x g for 10 min and then washed three times with 1xPBS, filtered using 40µm nylon mesh to remove cell clumps and last resuspended in HEPES-buffered RPMI containing 0.5% BSA. After the testicular single cell suspensions were obtained, two million cells were diluted in 2 ml of 1xPBS buffer and stained with Hoechst 33342 (5 µg/ml; Sigma) for 1h at 32°C. Before analysis, PI (2µg/ml; Sigma) was added to exclude dead cells. Finally, cell analysis and sorting were performed on a FACScalibur flow cytometer.
Instrument	Beckman coulter, MofloXDP
Software	summit5.3
Cell population abundance	After being dyed by the Hoechst 33342 (5 µg/ml; Sigma) for 1h at 32°C, the cells were sorted to different types (n,2n and 4n) for the following experiments through the a cell sorting system.
Gating strategy	According to the intensity of fluorescence stimulated by Hoechst 33342

☒ Tick this box to confirm that a figure exemplifying the gating strategy is provided in the Supplementary Information.
